# From liquid to solid: Exploring techniques, applications, and challenges of structured oils as fat replacements in food formulations

**DOI:** 10.1111/1541-4337.70163

**Published:** 2025-03-20

**Authors:** Ecaterina Savchina, Antonella L. Grosso, Petra Massoner, Ksenia Morozova, Matteo M. Scampicchio, Giovanna Ferrentino

**Affiliations:** ^1^ Faculty of Agricultural, Environmental and Food Sciences Free University of Bozen‐Bolzano Bolzano Italy; ^2^ A. Loacker Spa Unterinn Italy

**Keywords:** fat mimicking, glycerolysis, interesterification, oleogel, saturated fatty acids

## Abstract

Oil structuring is a strategy used to change the physical state of liquid oils to mimic the behavior of solid fats. In the past years, following the legislative bans on using partially hydrogenated fats and recommendations on limiting saturated fatty acid intake, oil structuring has become a fast‐developing research area. This review explores the current state of applications developed for oil structuring, considering the challenges and prospects. Processes such as direct and indirect oleogelation, as well as interesterification (acidolysis, alcoholysis, glycerolysis, and transesterification), are described, outlining the main factors governing them. The review also presents the potential applications and enhancement of the functional properties of structured oils in various food formulations. From the latest literature, the industrial applicability of structured oils is discussed. This work provides a well‐structured overview of the broad and diverse topic of fat mimetics and oil structuring, creating a solid base for a better understanding of the topic and spotting the challenges associated with their application.

## 1 INTRODUCTION

Solid fats—such as vegetable and animal fats—are essential to achieve the desired texture, spreadability, mouthfeel, and shelf life in food products. Solid fats contain high levels of *trans* and saturated fatty acids (SFAs), which have been associated with various health risks (Nettleton et al., 2017). In response to these findings, several legislative restrictions have been implemented in the European Union and the United States. Health authorities recommend limiting SFA consumption to no more than 10% of the total energy intake (WHO, 2023). As a result, industries face a demand for innovative approaches to reduce or eliminate *trans* fats and high levels of saturated fat content in their products.

Among several approaches, the substitution of solid fats with liquid oils seems a valuable alternative. This approach, however, results in technological problems due to the uneven lipid droplet distribution, which negatively affects the texture, the leavening of batters and doughs, the development of the gluten matrix, and the stabilization of gas bubbles during food preparation. Liquid oils, due to the low content of SFAs, do not provide the required physical properties in food products (Co & Marangoni, 2012). Moreover, the use of liquid oils also alters the sensory characteristics of the final products shortening their shelf life (Li et al., 2023).

Oil structuring is gaining popularity as a solution to this problem. Traditional fats can be exchanged with structured oil‐based systems as they can mimic solid‐fat‐like behavior and improve food product nutritional value or add functionalities. They have been investigated extensively as *trans* and saturated fat substitutes in a range of dietary items, such as baked goods, confectioneries, dairy, and meat products (Espert et al., 2023; Liu et al., 2019; Ma et al., 2023). Recent innovations in oil structuring have introduced novel methods and additives to improve the distribution, stability, and functionality of liquid oils in food products. Oleogelation techniques utilizing hybrid structuring agents, such as combinations of waxes, proteins, and polysaccharides, have shown promise in enhancing oil‐binding capacity while maintaining desirable sensory properties. Enzymatically modified oils, produced through lipase‐catalyzed interesterification and glycerolysis, enable the development of structured oils with tailored melting properties for improved textural and functional performance. These advancements not only support the reformulation of healthier lipid systems but also align with clean‐label and sustainability trends, increasing their potential for commercial adoption.

Despite the increased attention to the topic from both academia and industries, structured oils are yet to be successfully commercialized (Flöter et al., 2021). The complexity and variability of the proposed systems make product characterization, evaluation, and standardization challenging. Moreover, stability issues, classification challenges, and regulatory considerations further contribute to the slow commercialization of structured oils.

In the research community, a certain controversy exists in the application of terminology used to describe lipid modifications. In this manuscript, the authors adopted the terms “oleogel,” “structured lipids,” and “oil structuring techniques” as described in the following definitions:

Oleogel—a semisolid material represented by a structure based on liquid oil that can mimic the properties of solid fats. The process of forming an oleogel involves the use of structuring agents, which create a three‐dimensional network within the oil, thereby trapping it.

Structured lipids (SLs)—triacylglycerols (TAGs) that have been chemically or enzymatically modified to alter their fatty acid (FA) composition and/or the positional distribution of these FAs within the glycerol backbone to enhance the functional properties and nutritional profiles of these lipids. In this manuscript, glycerolysis reaction products will also be grouped with SLs.

Oil‐structuring techniques—an umbrella term, encompassing a broad range of methods for oil modification, resulting in structured oils such as oleogels or SLs, with the final goal of changing the physical properties of lipids (such as increasing solid fat content [SFC] or mimicking the solid‐fat‐like behavior) while aiming for enhanced functional properties and nutritional profiles.

Other works provide alternative definitions to the described terms. Flöter (2024) proposed to use the term “non‐triacylglycerol structuring” instead of “oleogelation” and referred to an oleogel as “non‐triacylglycerol structured lipid phase.” Pommella et al. (2020) adopted the term “oil texturing” and referred to oleogels with the broader term “organogels.” SLs can also be called designer lipids or tailor‐made fats (Jadhav & Annapure, 2021). Oil‐structuring techniques can also be referred to as lipid or fat structuring and intersect with oil modification, which includes interesterification, hydrogenation, and fractionation (Kellens & Calliauw, 2013). The products of implementing oil‐structuring techniques can also be addressed as fat mimetics (Cerqueira & Castro, 2023; Soleimanian et al., 2023).

This review aims to provide an overview of oil‐structuring techniques, reporting an updated state of the art on their application within food products. By focusing on understanding the main constraints related to oil‐structuring methods, this manuscript provides an answer to the question “Are the structured oils commonly adopted by the food industries, and why?” In the following chapters, different processes such as direct and indirect oleogelation, as well as interesterification (acidolysis, alcoholysis, glycerolysis, and transesterification), are described, outlining the main factors governing them. From the latest literature, the current research developments are discussed, emphasizing the enhancement of the functional properties of structured oils and their impact on the sensory characteristics in various food formulations.

## 2 OIL‐STRUCTURING METHODS

Several options can be found in the literature for classifying oil‐structuring techniques. Methods based on molecular characteristics of the structuring agents are used the most. Structuring agents can be classified into (i) low‐molecular‐weight organic compounds such as sterols, waxes, FAs, monoacylglycerols (MAGs), or lecithin and (ii) high‐molecular‐weight or biopolymeric compounds such as proteins and polysaccharides (starches, cellulose derivatives, and chitin) (Palla & Valoppi, 2024).

Oil‐structuring techniques can be also divided based on (1) the number of gelators used: (i) monocomponent and (ii) multicomponent gel (Okuro et al., 2020) and (2) the chemical nature of oleogelators: (i) lipid based, such as MAGs, diacylglycerols (DAGs), TAGs, FAs, fatty alcohols, γ‐oryzanol, phytosterols, ceramides, cocoa butter, lecithin, and waxes; (ii) nonlipid‐based, such as sorbitan and carbohydrate derivatives; and (iii) polymeric‐based structurants, such as proteins, cellulose derivatives, gums, and resins (Sagiri & Rao, 2020).

A recent review by Sabet et al. (2023) also proposed to classify oleogelation methods based on the energy consumed during the process (heat, electricity, and time inputs), dividing them into low‐, medium‐, and high‐input methods. Finally, oil‐structuring techniques can be also classified based on the formed lipid structure (crystalline or noncrystalline network, particle‐filled network, and polymeric strands) (Marangoni & Garti, 2018).

**FIGURE 1:**
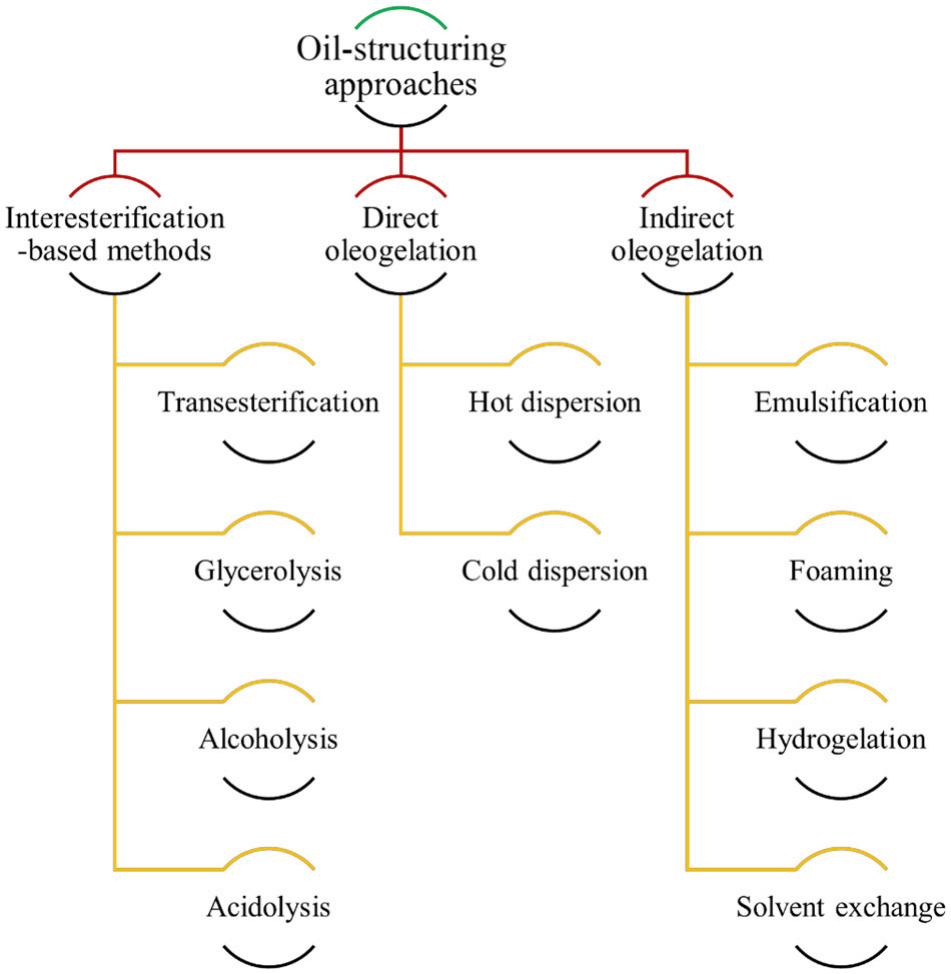
Classification of oil‐structuring methods.

In this review, the structuring approaches are organized based on the sequential processing steps involved in creating the structured oil systems. Figure 1 depicts this structure, dividing the approaches into direct and indirect oleogelation as well as interesterification‐based methods.

Besides the approaches reported in Figure 1, oleogel can be also formed at the latest stages of a production process. Namely, by incorporating air bubbles via simple whipping of the oleogels during the crystallization step, an aerated three‐dimensional network could be formed, called oleofoam or whipped oleogel (Callau, Sow‐Kébé, Jenkins, et al., 2020). Current research mainly focuses on oleofoams based on the oleogels formed through direct oleogelation methods. The oleogelator would then play the role of the foaming agent as well. For example, MAGs (Grossi et al., 2023); waxes (Borriello et al., 2025); FAs and fatty alcohols, such as stearic acid and stearyl alcohol (and their combinations) (Callau, Sow‐Kébé, Jenkins, et al., 2020); and behenyl alcohol and behenic acid (Callau, Sow‐Kébé, Nicolas‐Morgantini, et al., 2020) have been shown to be effective in creating physically stable oleofoams. The application of oleofoams allows the creation of new sensory experiences for the consumer and the formulation of low‐fat products with improved nutritional composition (Ribourg‐Birault et al., 2024).

Structuring methods should be chosen depending on the specific requirements of the application, such as the desired texture, stability, and functionality of the final product. Understanding the underlying principles and mechanisms of oil‐structuring techniques is crucial for developing innovative and high‐performing formulations.

### 2.1 Interesterification‐based methods

Interesterified fats have been introduced as replacements for *trans* fats in a wide range of food products such as spreads, bakery products, and confectioneries (Dijkstra & van Duijn, 2016; Mills et al., 2017; Puscas et al., 2020). Interesterification modifies the physical characteristics of fats, such as melting point and SFC, while preserving the favorable FA composition. The latest research covers multiple applications of interesterification for the production of plastic fats (margarines and shortenings), cocoa butter substitutes/equivalents, low‐calorie lipids, fats for clinical applications, human milk fat substitutes, and edible films (Moore & Akoh, 2017; Sivakanthan & Madhujith, 2020).

**FIGURE 2:**
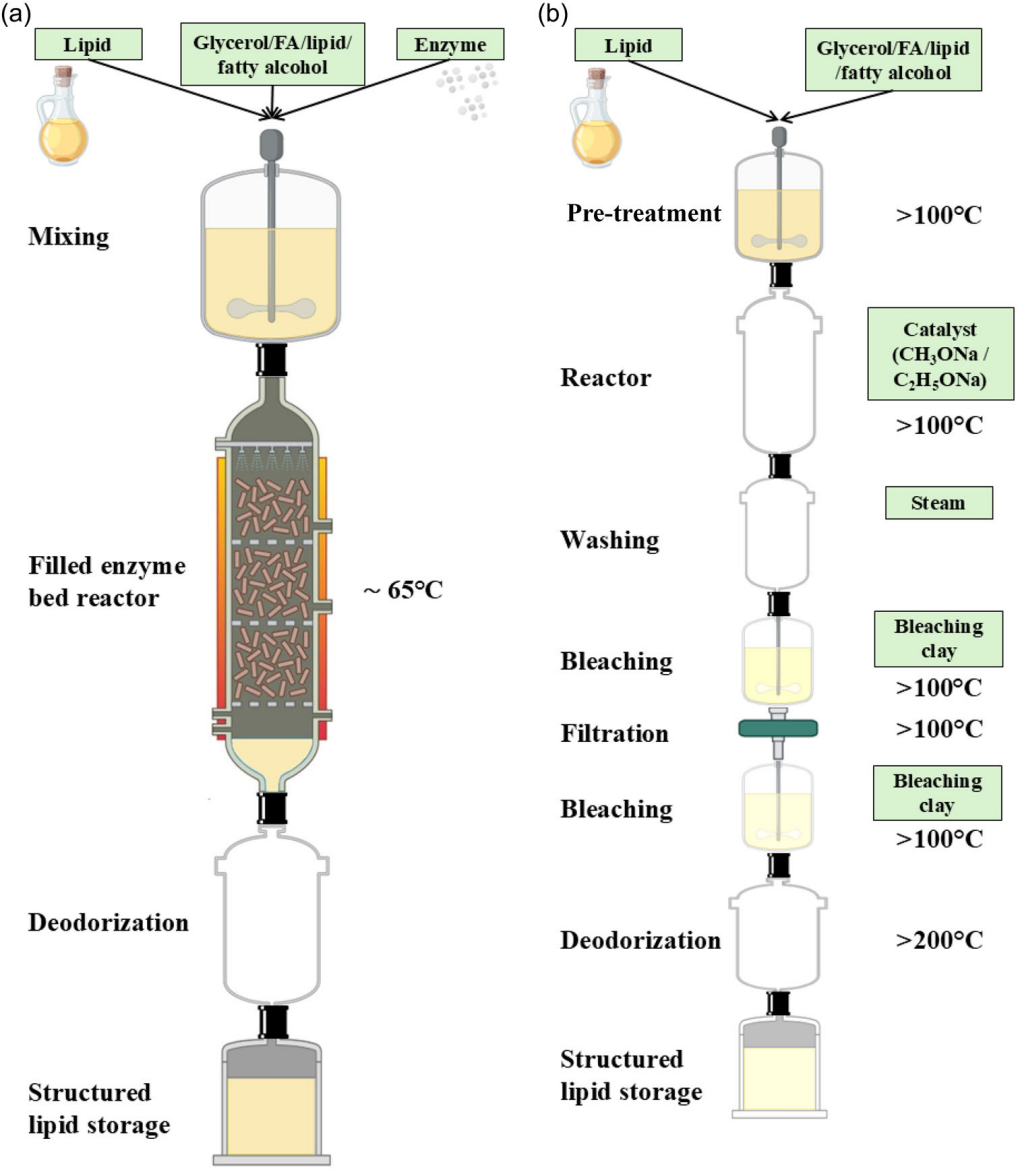
Enzymatic (a) and chemical (b) interesterification process.

Interesterification is a process that involves rearranging the FAs on the glycerol backbone of TAG molecules. Depending on the chemical compound reacting with TAG, interesterification reactions can be subdivided into four major categories: transesterification, alcoholysis, glycerolysis, and acidolysis. All mentioned techniques could be performed through either chemical or enzymatic pathways as shown in Figure 2. Chemical interesterification uses inorganic compounds as alkaline catalysts (sodium, potassium, and their alkoxides), while enzymatic interesterification employs enzymes of lipolytic activity, such as nonspecific lipases, sn‐1,3‐specific lipases, and FA‐specific lipases (Dayton, 2014). Figure 2a depicts the enzymatic approach, which usually follows a continuous manufacturing process, where the oil blend passes through a purification bed followed by an enzyme bed. Alternatively, a fluidized bed reactor or a stirred reactor (for a batch process) can be utilized (Basso & Serban, 2019). Chemical interesterification involves the use of alkaline catalysts added at levels of 0.05%–0.1% by weight to the purified oil or fat blend. The reaction occurs at elevated temperatures, typically between 90 and 150°C, under reduced pressure (Figure 2b). Chemically driven reactions are typically more affordable, quick, and reliable. However, the necessity of operating at elevated temperatures can accelerate oil oxidation processes and potentially yield 3‐monochloropropane‐1,2‐diol and glycidyl esters (Gibon & Kellens, 2014).

Transesterification is usually performed between solid fats (e.g., palm, shea, coconut) and liquid oils with a lower melting point but more appealing nutritional value. As a result, a new fat is formed with an altered TAG combination (changed molecular structure of the TAG), providing physicochemical properties of interest such as increased resistance to melting, enhanced digestibility, or improved oxidative stability (Flöter, 2024; Mensink et al., 2016). A similar principle is applied for acidolysis, where an FA reacts with a TAG to produce a new TAG.

Alcoholysis, instead, is a chemical reaction where an alcohol reacts with a TAG, becoming incorporated into the final product. The reaction results in new TAGs, partial acylglycerols, and glycerol. The obtained partial acylglycerides can be further esterified with free FA to achieve a higher yield of the TAGs with a tailored structure (Wei et al., 2020). Finally, glycerolysis is a process that structures liquid oils via high‐melting MAGs and DAGs through the conversion of TAGs. This is done by exchanging the hydroxyl groups of glycerol with the FA chains of a TAG. Even though glycerolysis is classified as a separate type of reaction, it is a type of alcoholysis reaction where glycerol takes the role of alcohol (Sivakanthan & Madhujith, 2020).

Reaction products of the enzymatically catalyzed interesterification reactions include newly formulated TAGs, partial acylglycerols, glycerol, FAs, and other minor compounds. Importantly, they are considered food grade, and their presence is usually allowed by the regulatory bodies. For instance, in the United States, MAG and DAG are classified as Generally Recognized As Safe (GRAS) by the Food and Drug Administration (FDA, 2014). This classification allows them to be used in food products without the need for premarket approval, provided they adhere to good manufacturing practices. In the European Union, mono‐ and diacylglycerides have been evaluated and approved by the European Food Safety Authority (EFSA). They are assigned as E 471, which indicates their status as a permitted food additive within the EU regulatory framework (Younes et al., 2017).

Moreover, by including favorable FAs such as medium‐chain fatty acids (MCFAs) or polyunsaturated fatty acids (PUFAs) into the TAG structure, SLs can provide nutritional benefits, such as lipids with lower caloric density and enhanced absorption of essential FAs (Alves et al., 2024; Hong et al., 2023). The designed SLs can help overcome obstacles in the absorption of long‐chain PUFAs in addition to facilitating the efficient use of the energy provided by MCFAs (Zhou et al., 2023).

Overall, oil structuring through interesterification techniques is a cost‐effective and sustainable approach. The established infrastructure for these processes allows integration and scalability in the food industry. The wide availability of raw materials ensures a consistent supply. Additionally, these methods enable the creation of fats with unique TAG compositions, which could be designed to achieve specific melting points, textures, and functionalities.

### 2.2 Direct oleogelation methods

**FIGURE 3:**
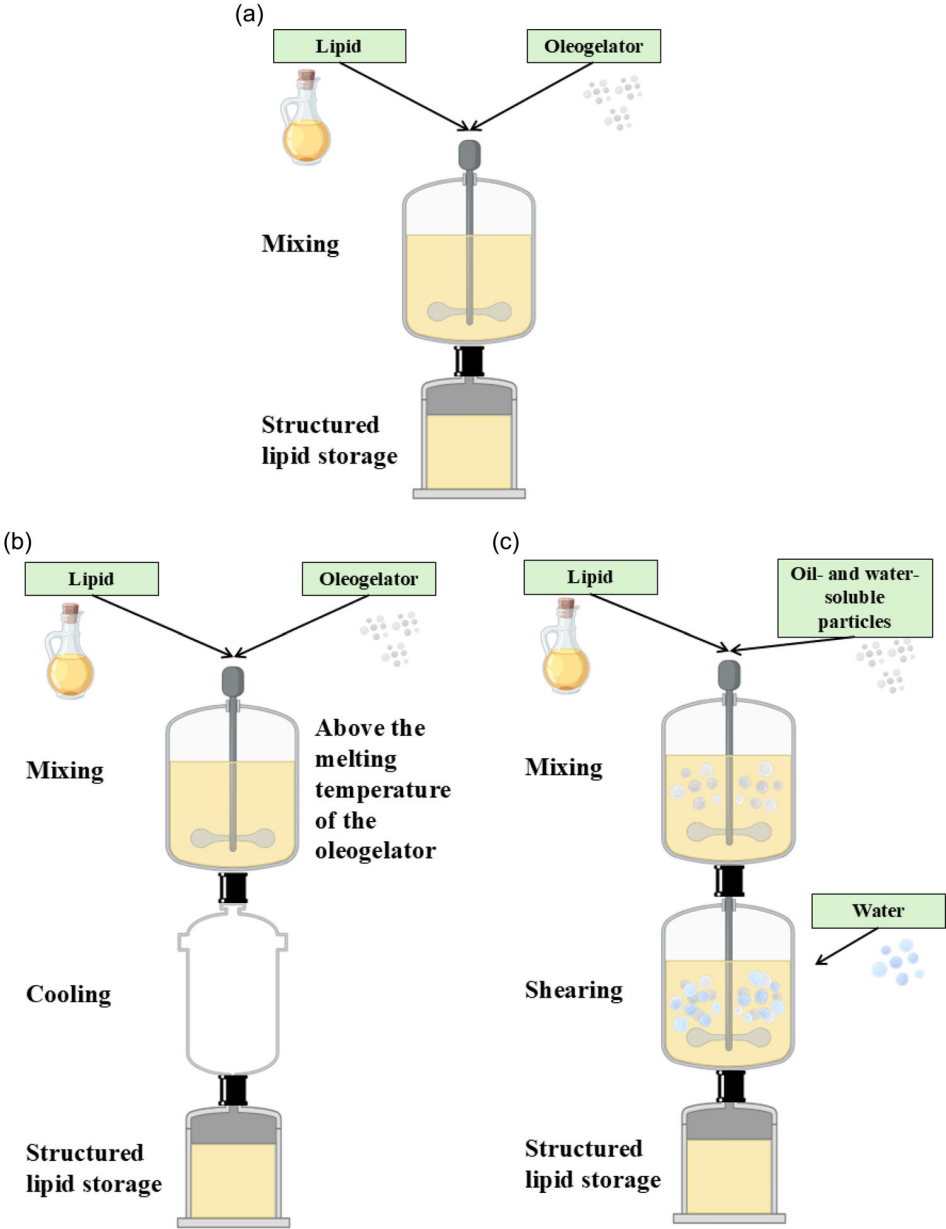
Cold direct (a), hot direct (b), and hot semidirect (c) oleogelation methods.

Direct oleogelation methods involve the straightforward dispersion of crystalline molecules in the oil phase. In other words, the approach includes the mixing of the lipid phase, represented by vegetable, animal, or marine oils, with a low‐molecular‐weight gelator or a mixture of such (Scharfe et al., 2020). Mixing the two components is achieved via homogenization, sonication, or standard mixing procedure in the presence of heating and cooling at different rates. Depending on the inclusion of the heating as a processing step, the direct oleogelation methods are subdivided into (i) hot direct method, in which the mixture components are warmed up to the melting point/glass transition of the gelator before mixing, and (ii) cold direct method, in which the components are mixed without preheating (Pinto et al., 2024). David et al. (2021) showed how by applying cellulose powders a structure via fiber entanglement can be obtained in rapeseed oil through the cold direct oleogelation method. If low amounts of water are added at the later stages of the oleogel production, a semidirect method is achieved (Valoppi et al., 2023). Due to the presence of water in the system, oleogelators that do not pose oil‐structuring properties when dispersed directly in oil can be used for oleogelation (Bodennec et al., 2016; Gao et al., 2023; Wang et al., 2022). Figure 3 shows a layout of the three processes. When water is incorporated in larger amounts, the systems are referred to as oleogel emulsions (Gaudino et al., 2019). They have been successfully applied at the industrial level by HI‐FOOD from CSM Ingredients, which uses natural vegetable fibers to create emulsions for SFA reduction applications.

The mechanical properties of the obtained structures highly depend on the nature of the oil, the gelator used, and the processing conditions. Among the gelators used for direct oleogelation, several options can be found, including waxes, MAGs and DAGs, lecithin, FAs, fatty alcohols, sterols, sterol esters, ethyl cellulose (EC), hydroxypropyl methylcellulose (HPMC), and many more. At industrial scale, waxes, MAGs, β‐sitosterol, and γ‐oryzanol are already frequently applied.

Wax serves as an umbrella term covering lipidic mixtures with varying proportions of wax esters (WEs), FAs, fatty alcohol hydroxides (FAOHs), hydrocarbons (HCs), and other minor components (Wettlaufer & Flöter, 2022). The composition and the physical attributes of wax can vary significantly based on factors such as its natural source, growth conditions, and processing techniques. Among the waxes commercially available and employed in patented formulations, it is possible to find carnauba (CBW), candelilla (CDW), sunflower (SFW), sugarcane (SCW), rice bran (RBW), and beeswax (BW). Due to the diverse sourcing together with its established history of consumption and handling simplicity, wax is highly regarded as a promising option for oil structuring. Research suggests that a significant portion of ingested edible wax may pass through the human body without being absorbed, reinforcing their appeal to be used in low‐calorie and health‐conscious products (Aguilar et al., 2012). Moreover, numerous scale‐up trials conducted in pilot plants have demonstrated the feasibility of using wax‐based oleogels in industrial settings, providing direct evidence of their suitability for large‐scale production (Palla & Valoppi, 2024).

MAGs are lipid compounds made of one FA esterified to a glycerol molecule. Usually, distilled MAGs based on SFAs can be utilized in oil structuring due to their ability to form gel networks within oil matrices, which depends not only on the degree of saturation and chain length but also on isomeric form. When dispersed in oil, small amounts of MAGs (5% for SFA based and 15% for unsaturated fatty acid [UFA] based) can self‐assemble into organized structures. MAGs are typically manufactured through the process of chemical glycerolysis described earlier. The use of UFA‐based MAGs requires higher amounts and often forms gels with lower hardness but brings the advantage of increasing the nutritional value of the food formulations. In addition, they exhibit a positive relationship with other gelators, including waxes (Chen et al., 2021; Sivakanthan et al., 2022). This can be used as a strategy to reduce the amount of added gelators while improving the shelf‐life characteristics of the fat ingredients obtained.

In addition, MAG and DAG blends are increasingly recognized for their potential as oil‐structuring agents in various food applications. Their unique chemical structures allow them to influence the physical properties of oils, making them suitable for creating stable emulsions and gels. Both MAG and DAG can act as crystallization modifiers in lipid systems. They influence the formation of crystalline networks within oils, which can enhance texture and stability (Wagner & Davidovich‐Pinhas, 2023).

The effects of these compounds on the crystallization behavior of TAGs have come to light since MAGs and DAGs often have greater melting and crystallization points. MAGs were shown to serve as effective nucleating agents in the crystallization process of fats and oils. They facilitate the formation of crystalline nuclei, which is the initial step in crystallization, allowing other molecules to aggregate around these sites and grow into larger crystals (Ribeiro et al., 2015). Saturated DAGs high in stearic acid were shown to accelerate cocoa butter crystallization (Chaiseri & Dimick, 1995). The use of partial acylglycerol blends thus was also shown to raise the SFC in oils (Vereecken et al., 2009).

The combination of β‐sitosterol and γ‐oryzanol (belonging to the sterols and sterol esters group) is one of the most studied gelators since they exhibit good oleogelation capabilities and, at the same time, do not bear unattractive fame of cholesterol among consumers. Moreover, they are commonly sold as dietary supplements. β‐Sitosterol together with other phytosterols is a byproduct of the edible oil refinery and wood pulp processing industries (Scharfe et al., 2020). γ‐Oryzanol is also obtained as a side stream—in this case, from the rice oil refining. Individually, both components have the ability to create crystalline particles within vegetable oils (Okuro et al., 2018).

EC is a polymer derivative composed of β‐d‐glucose monomers with partial replacement of free hydroxyl groups by the ethyl groups. It is considered safe to be used in food and pharmaceutical applications. When used as an oleogelator, EC can effectively structure oils to form oleogels with improved stability compared to traditional fats, especially during heating. The gel properties depend on the type and concentration of the oleogelator, the oil composition, and the processing conditions. In general, EC can form stable, thermoreversible oleogels with tunable rheological properties (Naeli et al., 2023).

Direct methods of oleogelation offer several benefits. In brief, these methods provide economically viable and, most importantly, naturally sourced, food‐grade solutions for structuring oils. Waxes are an example of effective and potentially economically attractive oleogelators, although they may have high compositional variability inherent to their natural source. Some waxes are also byproducts of the industry. In addition, the use of food hydrocolloids for the preparation of oleogels has gained increasing attention due to their biocompatible nature. However, a significant research effort is needed to overcome their intrinsic low solubility in the oil phase (Barroso et al., 2022).

From an industrial point of view, the direct oleogelation methods enable oleogel production without the need for new production lines, which leads to significant cost savings for manufacturers. Moreover, direct methods are economically feasible, sustainable, and an environmentally friendly choice. Another advantage lies in the gelling agent concentration needed to achieve gelation. The range of concentration for MAGs, waxes, and sterols/sterol esters typically falls between 2% and 5%, between 0.3% and 4%, and around 3%, respectively (Scharfe et al., 2020). This flexibility allows for fine‐tuning of texture and consistency in various applications, from spreads to confectionery.

### 2.3 Indirect oleogelation methods

The indirect methods of oleogelation consist of the preparation of the oleogel through intermediary steps rather than the direct mixing of the oil and the structuring agents (Palla & Valoppi, 2024). Frequently, one of these steps consists of the introduction of proteins and/or polysaccharides into the hydrophobic oil phase with the subsequent removal of the water phase (Feichtinger & Scholten, 2020). Adding these ingredients creates the gelling structure, which provides the desired system properties. The presence of these intermediary steps highlights the unique nature of this oleogelation method and its high complexity in comparison with other methods, such as direct oleogelation.

**FIGURE 4:**
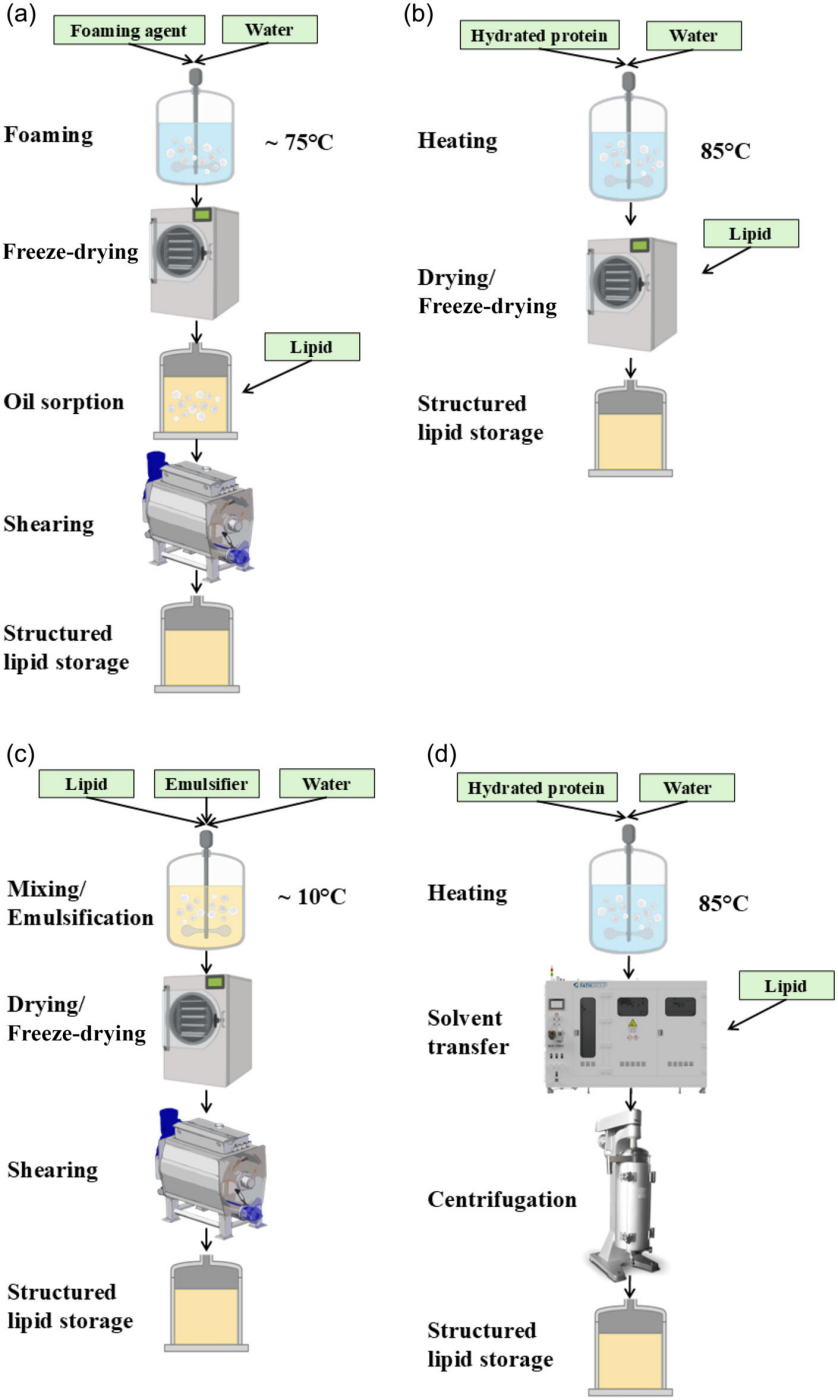
Indirect oleogelation methods: (a) foam‐templated; (b) hydrogel‐templated; (c) emulsion‐templated; and (d) solvent transfer approach.

The most widespread examples of this type of system are the foam‐templated, hydrogel, emulsion‐templated, and solvent transfer techniques (Figure 4). Briefly, the foam‐templated approach consists of the preparation of a foam with a subsequent freeze‐drying step. Afterward, the oil is poured into the obtained dried foam template until saturation is reached. The final amount of absorbed oil normally reaches over 90% and defines its absorption capacity (Feichtinger & Scholten, 2020). The hydrogel‐templated approach consists of the formation of an aqueous protein network and the subsequent removal of the water phase. The resulting structure is referred to as an aerogel before the addition of oil to create the oleogel. The method allows the removal of the aqueous phase by exchanging the water with an organic solvent of intermediate polarity (most likely ethanol), followed by either oven drying, freeze‐drying, or supercritical CO_2_ drying (Palla & Valoppi, 2024). In the emulsion‐templated approach, oleogels are produced using emulsions as the initial “templating” system. The method consists of the preparation of the initial template, normally an oil‐in‐water emulsion by the addition of an emulsifier and, alternatively, the addition of another ingredient for reinforcing the interface. This step is followed by dehydration, frequently performed by freeze‐drying. Finally, a shearing step is applied, leading to the formation of the oil‐continuous gel structure (Feichtinger & Scholten, 2020). To address the long drying stages inherent to this type of oleogelation method, highly concentrated emulsions can be prepared. By applying this approach, shorter drying times can be achieved (Li et al., 2023; Palla & Valoppi, 2024).

The solvent transfer method is different from the previously mentioned methods in terms of methodology and preparation steps. The technique consists of the formation of protein aggregates by heating a protein aqueous solution. Then, the water is replaced with oil by using an intermediate solvent and subsequent replacement of the solvent. The last stage involves getting a protein aggregated dispersion in oil and applying a centrifugation step to induce the gel formation (De Vries et al., 2015).

Generally, the ingredients used in the indirect methods are classified as high‐molecular‐weight biopolymers (proteins and polysaccharides). However, other types of compounds can be added to the mixture to promote the emulsifying properties, such as phenolic compounds (Santos et al., 2024). These ingredients have gained considerable interest due to their wide availability, food‐grade status, and general consumer acceptance (Palla & Valoppi, 2024). While proteins are normally considered to offer nutritional benefits, the polysaccharides used for these systems are categorized as nondigestible dietary fiber. Also, the combination of both macromolecules can be found very frequently (Sinha et al., 2024). HPMC, methyl cellulose, carboxymethyl cellulose (CMC), gelatin, different types of gums, and protein isolates from vegetal sources, among others, are the main examples of the macromolecules used for the indirect type of oleogelation (Jiang et al., 2022; Ma et al., 2023; Oh et al., 2019; Sinha et al., 2024; Wei et al., 2022).

## 3 CURRENT APPLICATIONS

Oil‐structuring methods have been tested to produce structured oils and assess their feasibility as substitutes for various fat sources, such as shortenings and butter, palm oil, cocoa butter, coconut oil, and animal fats. At the same time, commercial products containing oleogels are notably limited (Silva et al., 2022). The following sections present findings showing the potential of structured oils as healthier alternatives to traditional fats in various food applications. A summary is reported in Table 1.

**TABLE 1:** Recently published studies and patents on oil structuring applied to foodstuffs in the past 5 years.

Product	Method	Structuring agent	Substrate oil	Characteristics	Reference
Plastic fats (margarine, shortenings, and butter)	Direct and semidirect oleogelation methods				
	Hot direct oleogelation	Mixture of CDW and BW (3%, 5%, and 7%)	Soybean oil	Oleogels with a 3% wax concentration had similar or higher firmness than commercial stick margarines. Margarines made from oleogels with mixtures of both waxes had higher firmness than those made from a single wax.	Hwang & Winkler‐Moser, 2020
		CDW and CDW–BW in various ratios	Canola oil	Oleogels crystallized under dynamic conditions exhibited a more gradual stress drop or viscoplastic response, allowing them to better emulate the behavior of margarine during deformation.	Werner‐Cárcamo et al., 2024
		BW, alone or mixed with HC in a ratio of 9:1 (3% in the oleogel)	Sunflower oil	Margarines with an oleogel content of up to 30% were similar to the control in terms of firmness. An increase in the proportion of oleogels led to a decrease in the melting enthalpy of margarines.	Sobolev et al., 2023
		Saturated MAG (5%) combined with EC and CDW in different concentrations	High‐oleic safflower oil	Oleogels containing 4% EC, 5% MAG, and 3% CDW showed a viscoelastic modulus similar to a commercial product. The addition of CDW to the oleogels with EC‐MAG significantly increased their viscoelasticity and enhanced their properties compared to the commercial product.	Rodríguez‐Hernández et al., 2021
		MAGs (6% or 10%)	Sunflower, chia, flaxseed, soybean, and sesame oils	Oleogels were structurally stable with textural characteristics similar to spreadable margarines.	Dominguez et al., 2024
	Semidirect oleogelation	EC (7%) and GMS (0.5% or 1.0%)	High‐oleic safflower oil	Increasing the GMS concentration in the gelled W/O emulsions decreased the water droplet size, leading to increased gel elasticity and W/O emulsion stability due to a synergistic GMS–EC interaction.	García‐Ortega et al., 2021
	Indirect oleogelation methods				
	Emulsion‐templated approach	Soy protein isolate and XG	Sunflower oil	Oleogel hardness closely resembled that of butter and margarine.	Sinha et al., 2024
	Foam‐templated approach	Rice bran protein	Rice bran oil	The foams prepared at pH 7, 9, and 11 produced oleogels with more dense and uniform porous networks.	Wei et al., 2022
	Interesterification‐based methods				
	Transesterification method	Chemical interesterification	Tiger nut oil and palm stearin	Lower SFC and melting point in the interesterified oil, indicating improved suitability for margarine formulation.	Dong et al., 2023
		Enzymatic interesterification using immobilized lipase	Beef tallow with coconut oil	The interesterified oil with 40% coconut oil had the best properties for margarine production, with a low content of high‐melting‐point TAGs minimizing the “waxy” mouthfeel of beef tallow‐based margarines.	Cui et al., 2022
		Enzymatic interesterification using Lipozyme TLIM, lipase derived from *Thermomyces lanuginosa*	Refined rapeseed oil and fully hydrogenated rapeseed oil	The interesterified blends were suitable for creating zero‐*trans*, dialkyl ketone‐free margarines, retaining an optimal omega‐3 to omega‐6 ratio of 2.2, preserving tocopherols, and having improved melting characteristics and hardness scales that closely simulated those of commercial margarines. The enzymatically interesterified fats were softer and more homogeneous compared to non‐interesterified blends, which had a hard and brittle structure.	Danthine et al., 2022
		Enzymatic interesterification using Lipozyme TLIM lipase (*T. lanuginosus*)	Perilla seed oil with palm stearin	The obtained margarine exhibited several desirable characteristics, including a favorable omega‐3‐to‐omega‐6 ratio, improved melting properties, and a texture comparable to commercial margarines. The enzymatic interesterification process resulted in a softer, more homogeneous product with enhanced oxidative stability and preserved tocopherol content.	Dhiman et al., 2024
		Lipozyme TLIM	Mustard oil and palm stearin	The interesterified oil contained a higher total UFA content, compared to traditional shortening. The SFC of the SL was found to be higher than 10% at 20°C, which helps prevent oil exudation, and less than 2% at 40°C, avoiding a waxy mouthfeel. The SL had a high content of the β′ crystal polymorph and was satisfactorily spreadable at temperatures of 25 and 30°C, with ideal plasticity at 35°C, indicating that its functional properties were similar to those of commercial shortenings.	Manzoor et al., 2024
		Chemical interesterification	Ardeh oil and palm stearin	Interesterification allowed to decrease the SFC, slip melting point, and improve oxidative stability.	Tourchi Rudsari et al., 2019
	Glycerolysis	Lipase‐catalyzed glycerolysis using *Candida antarctica* lipase B immobilized on Immobead	Tigernut, cottonseed, peanut, olive, soybean, canola, rice bran, high‐oleic canola, sesame, and high‐oleic algal oils	Margarine formulated with glycerolysis‐structured oils, particularly tigernut oil, exhibited better performance in terms of plasticity and melting behavior compared to commercial margarine made with palm oil.	Nicholson & Marangoni, 2021
			Coconut stearin	Higher MCFA content, improved hardness, adhesiveness, and cohesiveness of recombined butter with 5% glycerolyzed oil.	Subroto et al., 2024
Shortenings applied in bakery products	Direct and semidirect oleogelation methods				
	Hot direct oleogelation	EC (4%)	Palm stearin and soybean oil (30%)	Oleogel had excellent air incorporation ability, creating shortening with fine crystals. Oleogel shortening application created breads with a superior volume to that of the commercial counterpart.	Ye et al., 2019
		HPMC (1.5%)	High‐oleic sunflower oil	Croissants with a lower saturated fat content, lower bite firmness, and a texture profile similar to croissants made with commercial shortening.	Espert et al., 2023
		HPMC and XG	Olive and sunflower oil	Enhanced rheological properties and thermostability with low oil losses (<10%) after 35 days of storage.	Bascuas, Morell, et al., 2021
		EC and MAGs (6% in sum)	Peanut DAG oil	Enhanced thermal resistance and oil binding capacity of oleogels with an improved distribution of crystals in the oleogel. The hardness of the cake decreased by 88.54% with the addition of MAG, showing compensatory effects.	Chen et al., 2023
		CDW (2 and 3%), RBW (2%), white BW (5%), yellow BW (5%), and MAG (5%)	High‐oleic rapeseed oil	Biscuits with 5% MAG oleogel exhibited similar properties to the palm oil biscuits and showed limited lipid migration.	Onacik‐Gür & Żbikowska, 2020
		Microcrystalline wax	Rapeseed oil	The biscuits baked in rapeseed oil with wax crystals were judged to be suitable replacements for palm fat in terms of processability, as well as analytical and sensory assessment.	Schmid et al., 2020
		MAG (6.6% w/w)	High‐oleic sunflower oil	The oleogel was an effective oil‐migration inhibitor for muffins, showing physicochemical properties very similar to those made with a commercial margarine.	Giacomozzi et al., 2023
	Indirect oleogelation methods				
	Foam‐templated approach	HPMC and GMS (double network)	Soybean oil	The double‐network oleogel reinforced by the GMS was similar to commercial fats in rheological properties. The oleogel successfully replaced 50% of the commercial butter in cookies and cakes.	Jiang et al., 2022
	Emulsion‐templated approach	Tp‐palmitate and citrus pectin	Camellia oil	Citrus pectin led to increased stability and viscoelasticity in the emulsions, resulting in oleogels with enhanced oil binding capacity and gel strength. The texture of the cakes showed an increase in hardness and chewiness compared to butter‐based cakes.	Luo et al., 2019
		XG, soy lecithin	Soybean oil	Replacing butter with oleogel reduced the hardness of the pound cake. Porosity of the pound cake increased, and air cells were much smaller in size and evenly distributed.	Hong et al., 2024
		Sodium caseinate and quercetin complexes	Sunflower oil	The cake batter produced with palm fat showed the highest viscosity and air incorporation. The quality parameters of the cakes produced with the oleogels were similar to those of the cake produced with sunflower oil after baking.	Santos et al., 2023
		Vanillin, Tween 60, chitosan, and acetic acid solution	Canola oil	Oleogel cookies exhibited a lighter surface color and higher redness attributed to the Maillard reaction. Oleogel cookies displayed similar hardness and crispness to shortening cookies.	Brito et al., 2022
		Canola protein isolate	Canola oil	Oleogel cakes displayed larger air bubbles, oil droplets, and protein aggregates and were darker in color compared to shortening cakes. Oleogel cakes exhibited lower hardness, higher springiness, and increased cohesiveness.	Tang & Ghosh, 2021
	Emulsion‐templated approach; foam‐templated approach; hot direct oleogelation	Emulsion‐templated: HPMC1500 or HPMC4000 (1%) and XG (0.6%). Foam‐templated: HPMC4000 (1.5%). Direct oleogelation: HPMC, MAG, sodium stearyl lactate, RBW, and BW (6%).	Direct method: corn oil 94%. Emulsion‐templated: 60% of corn oil. Foam‐templated: 6% of corn oil.	Cookies made with HPMC1500 emulsion‐templated approach had the highest hardness, while the lowest hardness was found in the cookies prepared with RBW and MAG. Cookies prepared with shortening, MAG, RBW, HPMC emulsion, and foam‐templated approach showed similar color properties, while sodium stearyl lactate and BW‐based oleogel resulted in a darker cookie surface.	Li, Wu, et al., 2021
	Interesterification‐based methods				
	Transesterification method	Chemically interesterified	IF1: 75% olive oil (OO), 15% *trans* fat, and 10% palm oil. IF2: 85% olive oil, 10% *trans* fat, and 5% palm oil.	Cakes produced with the fat blend containing 85% olive oil exhibited lighter surface color, proper symmetry, and a desirably curved surface compared to the control sample.	Kaçar & Sivri Özay, 2019
		Lipozyme TLIM	Palm stearin and rapeseed oil	The addition of interesterified oils (2%) notably elevated dough viscoelasticity and improved steamed bread qualities, by increasing the gluten strength and promoting its polymerization, thus surpassing the effects provided by physical blends.	Zhao et al., 2024
		Chemically interesterified	Ardeh oil (derived from sesame seeds) and palm stearin	The rice cookies produced with these shortenings exhibited superior textural characteristics, lower hardness, and better sensory attributes compared to those made with commercial shortening, which contained higher *trans* FAs.	Rudsari et al., 2021
	Alcoholysis/acidolysis	Interesterification of linseed oil and caprylic acid	Linseed oil	Resulting oil was lower in *trans*‐FA and had a higher oxidative stability as compared with the palm oil shortening. The cookies produced with SL obtained through acidolysis had a higher spread ratio, started expanding quicker, and had a higher set time than palm oil shortening‐based cookies.	Jadhav et al., 2022
Animal fats (meat and dairy products)	Direct and semidirect oleogelation methods				
	Hot direct oleogelation	EC (11% in oleogel), sorbitan monostearate (3.67% in oleogel), and BW (11% in oleogel).	Olive oil (44.39%), linseed oil (37.87%), and fish oil (17.74%)	The BW oleogel exhibited a more rigid, ordered, and brittle structure, while the EC oleogel was softer and more deformable, with conformational flexibility and higher thermal stability. The obtained oleogels had a high PUFA/SFA ratio and a low *n*‐6/*n*‐3 ratio, making them suitable as animal fat replacers for pork liver pâtés.	Gómez‐Estaca et al., 2019
		HPMC (3%)	Sunflower oil	Oleogels enhance the spreadability and reduce the mechanical strength of cream cheese.	Wang et al., 2024
		BW (7%)	Camellia oil	The overrun rate and the first dropping time of oleogel ice cream were higher than camellia oil ice cream, and the melting rate was lower than that of butter ice cream.	Jing et al., 2022
		CBW (6%, 8%, and 10%)	Soybean oil and peanut oil	The oleogel fat replacement (50% and 100%) reduced the melting rate of ice creams; however, it negatively affected the ice cream overrun.	Airoldi et al., 2022
	Indirect oleogelation methods				
	Foam‐templated approach	HPMC	Canola oil	The beef tallow replacement of HPMC oleogels results in lower cooking loss and a softer texture.	Oh et al., 2019
	Emulsion‐templated approach	Soy protein isolate and barley ß‐glucan	Linseed oil	Incorporating the oleogel in luncheon meat led to a reduction in hardness, gumminess, and chewiness. Luncheon meat showed a decrease in cohesiveness and resilience while maintaining unchanged springiness.	Ma et al., 2023
		Tween 80 (3.0%), Arabic gum (0.5%), and flaxseed gum (0.1%–0.9)	Soybean oil (70%)	Replacing the pork back fat with oleogels increased the protein contents and water‐holding capacity while decreasing the fat amounts in emulsified sausages.	Zhu et al., 2023
	Interesterification‐based methods				
	Transesterification method	Enzymatic interesterification using 1,3‐specific lipase enzyme	Palm kernel oil, caprylic acid (C8:0), and capric acid (C10:0)	Lower hardness values in frankfurters with 75% and 100% beef fat replacement with interesterified palm kernel oil compared to other groups.	Kılıç & Özer, 2019
	Glycerolysis	Enzymatic glycerolysis using Novozym 435	Peanut oil	The ice cream made with SLs showed a slower melting rate, a softer texture, and a more cohesive structure compared to the one prepared with unstructured oil.	Savchina et al., 2023
		Enzymatic glycerolysis	Shea, palm olein, tigernut, peanut, cottonseed, and rice bran oils	Oils with higher levels of SFAs and MUFAs displayed higher SFC at elevated temperatures and broader melting profiles with significantly higher melting points.	Soleimanian et al., 2023
		Enzymatic glycerolysis and molecular distillation to obtain high‐purity DAG oil	Palm oil/peanut oil blend	Promotion of partial coalescence of fat globules in the emulsion of whipped cream, leading to stable and preferable foam formation. Improved emulsion properties such as increased average particle size, surface protein concentration, partial coalescence of fat, and overrun during whipping when 0%–20% DAG was involved.	Liu et al., 2019
Cocoa butter substitutes (chocolate)	Direct and semidirect oleogelation methods				
	Hot direct oleogelation	Monoglyceride stearate, β‐sitosterol, lecithin, and EC (10 g/100 g)	Corn oil	Oleogel‐based chocolate showed improved physical properties. The β‐sitosterol + lecithin oleogel‐based chocolate displayed the lowest whiteness index and superior physical and bloom stability compared to the standard dark chocolate.	Li, Liu, et al., 2021
		β‐Sitosterol combined with oryzanol (2:3), stearic acid (1:4), and lecithin (4:1) (12% in oleogel)	Corn oil	The oleogel prepared with β‐sitosterol and oryzanol exhibited the strongest gel‐forming ability and the densest gel crystallization network among the three oleogels studied. The chocolate prepared with (cocoa butter and oleogels at a 1:1 ratio) showed similar texture, crystal structure, and rheological properties to dark chocolate.	Sun et al., 2021
		SFW and polyglycerol stearate	Hazelnut oil (oil:gelator 90:10)	The sample prepared with oleogel was stiffer and melted slower compared to the control sample. No fat bloom was observed in the samples during a 15‐day storage period with temperature fluctuations.	Yılmaz & Öz, 2024
	Indirect oleogelation methods				
	Emulsion‐templated approach	HPMC and XG	Extra virgin olive oil, refined sunflower oil, and virgin flaxseed oil	Spreads of olive and sunflower oleogels exhibited greater firmness and spreadability compared to coconut fat spreads. Complete replacement of coconut butter with oleogel resulted in less homogeneous spreads, indicating the importance of the balance between the two components for structural integrity and sensory attributes.	Bascuas, Espert, et al., 2021
		HPMC (1%)	Sunflower oil (47%)	The replacement of cocoa butter with oleogel had a significant impact on the consistency and hardness of the systems, with values diminishing as the oleogel percentage increased.	Alvarez et al., 2021
	Foam‐templated approach	HPMC (1% w/w)	Refined sunflower oil	Increasing the oleogel proportion decreases its viscoelasticity and thermal parameters. From a technological point of view, a replacement level of 50% was appropriate.	Alvarez et al., 2021
	Interesterification‐based methods				
	Transesterification method	Enzymatic interesterification	Palm olein, fully hydrogenated palm oil, and palm kernel oil	The best‐obtained characteristics of the SL were observed when cocoa butter substitute was produced from a combination of palm kernel oil and enzymatically modified fat with a ratio of 4:6. Chocolate made with the oleogel showed desired properties in terms of hardness and fracturability without the need for tempering.	Zhang et al., 2020
		Enzymatic interesterification using Lipozyme RMIM	Cinnamomum seed oil and fully hydrogenated palm oil	The SL exhibited a ball‐like, well‐distributed, and nearly round crystal microstructure with a smaller crystal size. The SFC of the SL up to 38.47% at 25°C was beneficial for improving spreadability in confectionery products and baked goods.	Ma et al., 2019
			Palm kernel stearin, coconut oil, and fully hydrogenated palm stearin	SL blends 60:10:30 and 70:10:20 (palm kernel stearin:coconut oil:fully hydrogenated palm stearin) showed similar melting ranges, melting peak temperatures, and SFC to the commercial cocoa butter alternative.	Dong et al., 2023
		DF IM, sn‐1,3‐selective lipase derived from *Rhizopus oryzae*	Illipe butter stearin and palm mid‐fraction	Decreased contents of POP (palmitoyl‐oleoyl‐palmitic) and StOSt (stearoyl‐oleoyl‐stearic), while the content of POSt (palmitoyl‐oleoyl‐stearic) increased. The slip melting point, SFC curve, melting peaks, and melting completion temperatures of the interesterified blends, particularly the 60:40 blend, were found to be similar to those of commercial cocoa butter. The microstructure of the interesterified fat was found to crystallize into the same polymorphic form as cocoa butter, indicating their full compatibility.	Sonprasert et al., 2022
		Chemical interesterification	Palm kernel stearin, coconut oil, and fully hydrogenated palm stearin	Blends with 60%–70% palm kernel stearin showed melting characteristics similar to commercial cocoa butter alternatives, melting almost completely at body temperature. The SLs demonstrated improved crystallization behavior, with a crystal morphology similar to that of commercial cocoa butter alternatives, maintaining a desirable polymorphic form, although with smaller crystal sizes.	Ornla‐ied et al., 2022
	Alcoholysis/acidolysis	Enzymatic acidolysis	Grapeseed oil	The decrease in saturated fat had a negative effect on the rheological properties of the spreads, with a reduction in viscosity and texture parameters. The spread with 75% substitution of vegetable fat by structured TAG showed a severe reduction in rheological properties that affected the product spreadability.	de Souza Correia Cozentino et al., 2022

Abbreviations: BW, beeswax; CBW, carnauba wax; CDW, candelilla wax; DAG, diacylglycerol; EC, ethyl cellulose; FA, fatty acid; GMS, glycerol monostearate; HC, hydrocarbon; HPMC, hydroxypropyl methylcellulose; MAG, monoacylglycerol; MCFA, medium‐chain fatty acid; PUFA, polyunsaturated fatty acid; RBW, rice bran wax; SFA, saturated fatty acid; SFC, solid fat content; SFW, sunflower wax; SL, structured lipid; TAG, triacylglycerol; UFA, unsaturated fatty acid; W/O, water–in–oil; XG, xanthan gum.

### 3.1 Spreadable fats and butter/margarine substitutes

Spreadable fats can be successfully produced by interesterification. The technique can yield favorable properties for fat spreads mimicking the characteristics of SFA‐rich fats but maintaining a high concentration of UFAs (Danthine et al., 2014). In recent years, several studies have shown promising results by applying interesterification methods to improve the SFC, melting behavior, spreadability, and crystal morphology of spreadable fats (De Martini Soares et al., 2013; Dong et al., 2023; Nicholson & Marangoni, 2021; Pande & Akoh, 2013). Interesterification‐based approaches usually include adjusting the ratios between oils of various origins and solid fats containing high‐melting TAGs to create plastic fats (Motamedzadegan et al., 2020). By optimizing these variables, it becomes possible to achieve the desired melting behavior, avoid a waxy mouthfeel, and improve the oxidative stability of the products (Cui et al., 2022; Tourchi Rudsari et al., 2019).

Manzoor et al. (2024) developed a nutritionally beneficial spreadable fat from mustard oil and palm stearin‐based interesterified lipid. As a result, an increase in UFAs and a decrease in tocopherol content were observed. In another work, the transesterification of beef tallow with coconut oil made it possible to obtain a spreadable fat with a more compact texture, improved color, and minimized waxy mouthfeel when compared to a commercial counterpart. Due to the low content of high‐melting TAGs, the sample with 40% coconut oil allowed to decrease the “waxy” mouthfeel perception (Cui et al., 2022).

Through glycerolysis, a margarine‐like product with improved plasticity was obtained from tigernut oil. An increase in the SFC from 8% to 34% at 5°C was observed in the oil after glycerolysis (Nicholson & Marangoni, 2021). It was also shown that glycerolysis reaction products contributed to the development of a distinctive aroma well‐liked by the panelists when added in lower concentrations (5%) (Subroto et al., 2024).

Among structured oils, SLs have the largest presence in various food market segments (Sivakanthan & Madhujith, 2020). The introduction of hard butter for chocolate by Fuji Oil in the mid‐1980s marked the first industrial application of lipase interesterification technology. This innovation not only enhanced the texture and stability of chocolate products but also set a starting point for the use of SLs for other food applications. SLs are frequently developed for business‐to‐business (B2B) applications in the food industry before being applied for business‐to‐consumer (B2C). For instance, a B2B business might produce a fat ingredient (plastic fat) for a B2C company that sells dairy products or baked goods at retail. Since these products are usually more expensive due to the additional value, they are positioned in niche markets as opposed to commodity industries (Kleiner & Akoh, 2018). Today, companies like ADM, Lipsa, and Bunge Oils have successfully applied enzymatic interesterification to produce modified oils and margarines with improved functionality and health profiles, addressing consumer preferences for *trans*‐fat‐free options. They can be used as SFA‐free shortenings and *trans*‐free margarines alternatives, such as Crokvitol, Regal, NovaLipid, and SansTrans (Kim & Akoh, 2015). While these products are intended for all‐purpose applications, some shortenings have a more specific designation of use. The oil used as a substrate for the production of many of the products herein mentioned is soybean. In addition, interesterification reactions are also used to produce DAG oil, which can also be found on the market. DAG oil represents a high fraction of DAGs (>80% as opposed to less than 10% in traditional vegetable oils) (Lo et al., 2008). DAGs were linked to a number of health benefits. In addition to elevated levels of high‐density lipoprotein cholesterol, these include decreased serum TAG levels, calorie reduction, body fat stores, immune function improvement, low‐density lipoprotein cholesterol, and total cholesterol (Feng et al., 2022; Guo et al., 2020).

Direct oleogelation methods using waxes allow for mimicking the firmness of commercial margarine but are not able to provide a similar short‐time elastic response (Hwang & Winkler‐Moser, 2020). The latter can be achieved through dynamic crystallization (Werner‐Cárcamo et al., 2024). For achieving a better sensory profile of the obtained products, a partial substitution of the fat with oleogels has been proposed (Sobolev et al., 2023). It has been reported that the application of different waxes or a combination of various oleogelators improves the rheological behavior (viscoelasticity and recovery rate of elasticity) of oleogels (García‐Ortega et al., 2021; Marangoni et al., 2020; Rodríguez‐Hernández et al., 2021). For instance, a 3% wax oleogel margarine with 25% CDW and 75% BW had significantly higher firmness than margarine with 100% CDW or 100% BW (Hwang & Winkler‐Moser, 2020). Oleogels formed by combining distilled MAGs of vegetable FAs and EC with or without CDW exhibited mixed elastic recoveries of more than 100%. This value was not achieved by the use of single oleogelators. Moreover, the results showed that the rheological behaviors of oleogels were strikingly similar to those of commercial edible shortenings (Rodríguez‐Hernández et al., 2021).

Dominguez et al. (2024) reported the highest hardness and oil‐binding capability as well as superior nutritional quality for the unrefined flaxseed and chia oleogels structured with monostearin. The unrefined nature of the oils had a positive effect on fat crystallization. Furthermore, the use of SFA‐rich MAG allowed an improvement in the oxidative stability of the final products. The produced spreadable fats reported characteristics similar to commercial margarines, thus showing that, by the application of MAG alone, great results could be achieved as well. Examples of indirect methods demonstrate the possibility of mimicking margarine hardness successfully and even surpassing conventional spreadable fats in terms of viscoelasticity (Patel & Dewettinck, 2015; Sinha et al., 2024). Nevertheless, the obtained texture and sensory properties are largely dependent on the oleogelator and the type of indirect method employed. Furthermore, some studies have shown that indirect types of oleogels present a superior capacity to mimic commercial shortenings than direct methods of oleogelation. For instance, Li, Wu, et al. (2021) reported *G*ʹ value of shortening, made with HPMC‐based emulsion‐templated and HPMC‐based foam‐templated oleogels, significantly higher than the one obtained with MAG, stearyl lactate, RBW, and BW oleogels. The authors were able to conclude that the HPMC‐based indirect method of oleogelation was able to form a stronger gel network with a greater ability to resist deformation in comparison to direct‐method oleogels, obtaining a product with rheological characteristics comparable to commercial shortening. Additionally, Sinha et al. (2024) claimed the possibility of producing an oleogel via the emulsion‐templated method by using soy protein isolate and xanthan gum (XG) as a trans‐free and low‐saturated alternative solid fat, with a hardness similar to commercial butter and viscoelasticity better than the solid fats.

### 3.2 Shortening substitution for bakery and confectionery applications

Interesterification shows several potentials in the development of fats suitable for substituting shortenings. Currently, on the market, Ultra Blends offers shortenings not only for baked goods like cookies, crackers, biscuits, and flatbread/tortillas but also for dairy fat replacement and popcorn preparation, opening up larger possibilities for the interesterified fat applications. Enzymatically interesterified rapeseed oil has been shown to significantly improve dough viscoelasticity, the structural integrity of the gluten network, and the quality of steamed bread (Zhao et al., 2024). In literature, glycerolyzed vegetable oils have been used to produce margarine and peanut butter, offering similar textural properties to those made with traditional fats (Nicholson & Marangoni, 2020, 2021). Depending on the oil used for the interesterification, the sensory profile of the obtained product can be affected in both positive and negative ways (Jadhav et al., 2022; Kaçar & Sivri Özay, 2019).

Besides interesterification, direct oleogelation methods prove to be promising in the field of shortening substitution for bakery and confectionery applications. It was shown that by using XG and HPMC, softer and more compact bakery products are produced (Bascuas, Morell et al., 2021). Research on high‐oleic soybean oil oleogels revealed that they provided similar lubrication effects and developed comparable gluten networks as traditional shortening. The study found that doughs made with MAG and RBW oleogels showed good tolerance during mixing (Zhao et al., 2022). The use of MAG‐based oleogels tends to reduce the hardness, thus improving the sensory assessment outcomes of the baked goods and playing an important technological role in decreasing the oil migration (Giacomozzi et al., 2023). Chen et al. (2023) also observed that the properties of MAGs decreased the hardness of oleogel‐based sponge cake, which allowed for diminishing the negative impact of EC on the rheological properties of EC–MAG oleogel prepared using peanut DAG oil as a substrate. A limitation of the oil migration was reached through the application of DAGs present in the DAG oil.

Ye et al. (2019) used EC100 at 4% (w/w) in conjunction with base oil (30% degree of saturation by combining palm stearin and soybean oil) to create the shortening, which was later applied in bread formulation as an antifirming agent. As a result, breads showed stable and soft texture and a higher specific volume, when compared to the commercial counterpart due to the increased air‐incorporating ability.

**FIGURE 5:**
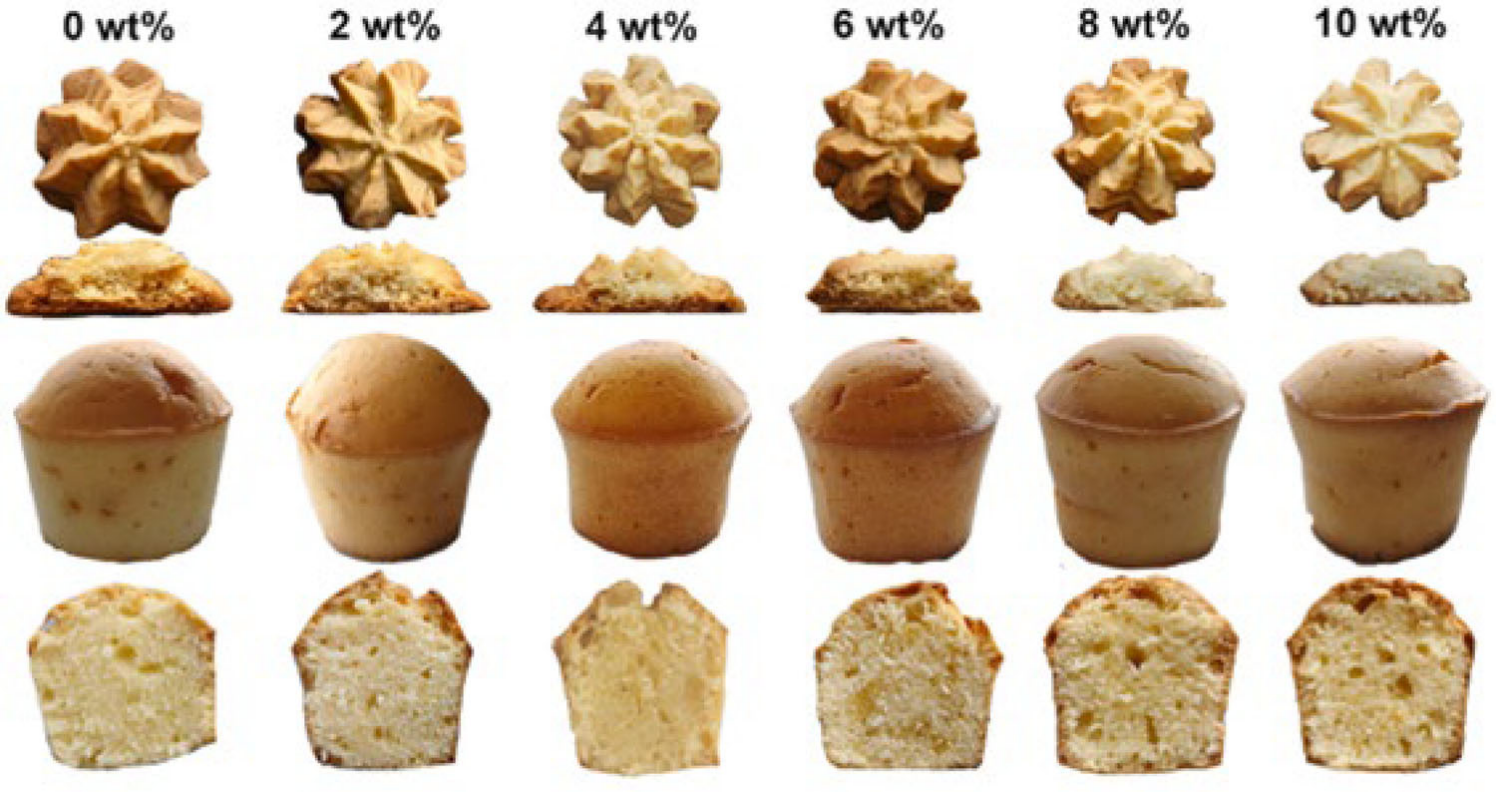
Crust and crumb visual appearance of cookies and cakes prepared by adding 50% double‐network oleogels. Reproduced from Jiang et al. (2022).

Several studies proved that oleogels produced via indirect methods are potential shortening substitutes in baked goods. In recent research, the physical properties and thermal sensitivity of foam‐templated oleogels were similar to shortenings available on the market (Wei et al., 2022). The authors showed the potential to substitute 50% of commercial butter in cookies and cakes with a foam‐templated HPMC‐based replacer. Moreover, the texture of baked products was adjusted by optimizing the glycerol monostearate (GMS) crystal network to enhance its effect on oleogels, as shown in Figure 5 (Jiang et al., 2022). With the addition of the GMS, the hardness, fracturability, and chewiness increased in both cookies and cakes. On the other side, the cohesiveness, springiness, and gumminess decreased.

Emulsion‐templated oleogels with increased citrus pectin concentration formed oleogels with high gel strength, good heat stability, and high antioxidative activity, comparable to butter (Luo et al., 2019). However, when applied to cake batter, the resulting products were harder and had lower consumer acceptance.

A study by Santos et al. (2024) showed similar results for different fat sources applied for pound cake preparation using the oleogel emulsion‐templated approach. Cakes prepared with palm fat, sunflower oil, sodium caseinate‐based oleogel, and sodium caseinate‐quercetin‐based oleogel exhibited different properties. However, the quality parameters of the cakes made with oleogels were like those made with sunflower oil.

### 3.3 Animal fat substitutions in meat and dairy products

Interesterification‐based methods are widely applied for animal fat substitutions in meat and dairy products. Transesterified fats could be directly applied to sausages and frankfurters due to the tailored melting behavior and hardness (Kılıç & Özer, 2019). In the case of glycerolysis, besides the change in the thermal properties of the obtained fats, newly formed mono‐ and diacylglycerols improve the emulsifying properties. This attribute can be beneficial to the bakery products as described earlier and can be used in products commonly containing added emulsifiers. Recently, an application of glycerolyzed peanut oil was proposed to produce an animal fat‐free ice cream formulation, which allowed for increasing the emulsion stability, slowing down the melting of the ice cream, and reducing its hardness, when compared with vegetable oil‐based counterpart (Savchina et al., 2023). Increased hardness is considered an undesirable characteristic in ice creams since it is related to the size of ice crystals and the structure of air cells. Larger ice crystals and a higher ice phase volume produce harder and more icy ice cream, which is perceived negatively by consumers.

Animal fat mimetics were studied by applying glycerolyzed oils in combination with EC (Soleimanian et al., 2023, 2024). For instance, palm olein and shea olein systems showed an increased SFC due to their high SFA concentration, indicating good structuring properties. In the presence of EC, the authors succeeded in formulating an oleogel that would function as adipose tissue with good mechanical and melting characteristics.

Direct methods have been applied to produce a wide range of meat and dairy products. For instance, wax‐based oleogels were used for ice cream preparation. The panelists reported positive feedback on the sensory attributes in case of partial replacement of fats with oleogel (Airoldi et al., 2022; Jing et al., 2022). On the other side, in the case of EC and HPMC applications, negative impacts were observed on the sensory characteristics of the products (Gómez‐Estaca et al., 2019; Wang et al., 2024).

Emulsion‐ and foam‐templated approaches are more commonly studied when replacing animal fats. For instance, foam‐templated oleogels used in sandwich cookie creams had physical characteristics like commercial products at 50% and 75% replacement levels for creams containing 40% fat content. This substitution resulted in creams with lower saturated fat content, long‐term oil stability, less sticky texture, and rheological behavior like the commercial benchmark product (Tanti et al., 2016).

The replacement of beef tallow with foam‐templated HPMC oleogels in meat patties significantly reduced SFA levels (from 42% to 15%), lowering the cooking loss and producing a softer texture without sacrificing the sensory properties (Oh et al., 2019). In recent studies, sausages and luncheon meats were produced with emulsion‐templated oleogels, resulting in a more compact texture, reduced cooking loss, and gumminess, but lower scores in sensory evaluation (Ma et al., 2023; Zhu et al., 2023).

### 3.4 Cocoa butter substitutes for chocolate and confectionery products

Cocoa butter substitutes are vital for the food industry as a cost‐effective and sustainable alternative to the limited and expensive natural cocoa butter.

The first example of the application of an interesterification technique for the development of a cocoa butter‐like fat was found back in the 1980s when Unilever and Fuji Oil company patented an approach using lipase from *Rhizopus niveus*, immobilized by adsorption onto diatomaceous earth (Coleman & Macrae, 1979; Takaharu et al., 1981).

In recent years, coconut oil, palm olein, and palm kernel oil fractions have been used in transesterification processes with the formation of βʹ polymorphs. From that, bloom‐resistant chocolate formulations, which do not require a tempering process, can be developed (Sivakanthan & Madhujith, 2020).

At the same time, other approaches include a combination of lauric and non‐lauric oils, which are then hydrogenated and subsequently interesterified (Cruz & Alvarez, 2012; Jung et al., 2006). The patented work of CJ CheilJedang Corp describes the separation of the solid phase of interest from the interesterification reaction products through the distillation of FAs, ethyl esters, MAGs, and DAGs, as well as the fractionation of the reactants (Ji‐Hyun et al., 2009).

Direct oleogelation methods can also be effective in forming structured oils to be used in chocolates. The product shows enhanced thermal stability when waxes and polyglycerol stearate are both applied during oleogelation (Yılmaz & Öz, 2024). Moreover, the sensory characteristics of the chocolates are also improved making the chocolates less hard and glossy.

β‐Sitosterol and oryzanol have been also actively explored in the latest literature. Studies showed that the obtained chocolates proved superior physical stability and resistance to blooming (Li, Liu, et al., 2021; Sun et al., 2021).

Oleogels produced by indirect methods have also shown good potential in terms of cocoa butter substitutions. A study using the foam‐templated approach noted that the prepared oleogels had higher thermal stability than the cocoa butter. In the cocoa butter/oleogel blends, an increase in the proportion of the oleogel (up to 70%) weakened the crystalline network of cocoa butter as reflected by the rheological, thermal, and textural parameters (Alvarez et al., 2021). A similar study using the emulsion‐templated approach found that HPMC‐based oleogels could partially replace cocoa butter (50%), reducing the SFA content by 39% in chocolate while maintaining optimal sensory properties. Ultimately, when preparing chocolate by using HPMC‐based oleogel:cocoa butter blend (50:50), the final product presented a very similar appearance to the cocoa butter control chocolate. Nevertheless, different concentrations of HPMC used for the oleogel formation impacted the textural properties. For instance, higher concentrations of HPMC (1.5% and 2%) resulted in softer samples, while a lower HPMC concentration (0.5%) resulted in flavor and textural properties similar to the cocoa butter control (Espert et al., 2021).

At the industrial level, Perfat Technologies has declared its plan to apply oleogels in chocolate pralines. Although the producer has not yet specified which oleogelation system was applied, several patents were published using the emulsion‐templated approach (Valoppi et al., 2024, 2022).

It is worth underlining that most of the available information is drawn from empirical studies. The complexity of the preparation procedure remains the main reason why these substitutes are still not widely applied in the food industry (Li et al., 2023).

### 3.5 Special applications of SLs

Since a long time ago, Stepan Specialty Products LLC has been manufacturing medium‐chain triacylglycerides (MCTs) from glycerol, caprylic, and capric FAs. Their commercial product, NEOBEE MCTs, can be used as an excipient in pharmaceutical applications for both humans and animals, as well as in medical nutrition products. SLs are also used by Abbott Laboratories to enhance the absorption of specific FAs in their Abbott Nutrition for Healthcare and Professionals line. By using SLs from interesterified marine oils, the product Vital AF Cal is a therapeutic nutrition solution designed to manage inflammation (Kleiner & Akoh, 2018).

For dietary management where fat absorption is impaired, NUTRITIA offers an unflavored MCT oil that consists entirely of 100% MCT oil. Similarly, Nestlé Health Science markets MCT Oil, which serves as a supplemental fat source for individuals with difficulties in digesting conventional fats. These products are commonly made from modified coconut and/or palm kernel oil. PromoPharma provides Dimagra MCT Oil, ideal for low‐calorie diets. MegaChem offers various grades of MCTs derived from kernel palm oil or coconut oil, depending on the ratio of caprylic (C8) to capric (C10) acids. The available grades range from 60% to 99% C8 content. MCTs are beneficial in baked goods, confectionery, pasta, and sports nutrition due to their stability and neutral flavor (Lee et al., 2022). In addition, MCT oils are used as glazing and sealing agents, for example, for dried fruits. In the consumer market, MCT oils are also available as ingredients for ketogenic diet products. Examples include Kanso by Dr. Schär, which features MCT tomato or champignon paste and MCT cacao bars, as well as the MCT Line from Keforma for puddings, chocolates, and cookies.

Delta is a series of healthy oils from Bunge Oils made to improve the nutritional value of foods. An oil enriched with omega‐3 FAs is a noteworthy product in this category. It is made from SLs, which include stearidonic acid, MCTs, and canola oil. Another example is SLs manufactured from high‐oleic canola, MCTs, and phytosterols that are specifically designed to help the regulation of cholesterol levels.

SLs are also useful in providing infants with specialized nutrition. By placing palmitic acid at a specific position in TAGs, Betapol (Bunge Oils), which is well‐known for its OPO (P: palmitic acid and O: oleic acid) structure, simulates human breast milk in infant formulae and improves absorption. Similarly, InFat, a vegetable structured oil offered by Advanced Lipids, positions palmitic acid at the sn‐2 position for optimal metabolism in infants. By mimicking the intricate lipid composition of human milk, these SLs are intended to enhance digestive health and nutrient absorption. Danone's innovations (Nuturis) further emphasize this trend, creating infant formulas with larger lipid droplets coated with milk phospholipids, other polar lipids, (glyco)proteins, and cholesterol to support growth and nutrient uptake (Breij et al., 2019). These advancements improve infant nutrition by closely resembling the composition of human milk fat.

## 4 OIL‐STRUCTURING EFFECT ON SENSORY PROFILES OF FOOD PRODUCTS

From an industrial applicability perspective, the sensory characteristics of foods play a crucial role in the development, acceptance, and success of new products and technologies. Oil structuring can significantly influence sensory attributes, including appearance, color, taste, body or texture, mouthfeel, odor/flavor/aroma, and overall consumer acceptability.

The comparison of sensory attributes of structured oils prepared using different methods remains largely underexplored. To date, no study has comprehensively contrasted the sensory properties of structured oils obtained through indirect methods, direct oleogelation, and interesterification‐based approaches. However, a recent study by Li et al. (2021) examined the effects of indirect (emulsion‐templated and foam‐templated) and direct (hot direct oleogelation) methods on the texture and sensory properties of structured oils. The study also evaluated the potential of these oils as shortening replacements in cookie production. The findings revealed that structured oils produced by direct methods, particularly with MAGs and RBW, achieved sensory acceptability comparable to shortening. In contrast, structured oils prepared using indirect methods (emulsion‐ and foam‐templated approaches based on HPMC) exhibited the lowest sensory performance, rendering them unsuitable for cookie production. These sensory results aligned with the textural properties of the prepared cookies. Cookies made with indirect‐method oleogels exhibited the highest hardness—an undesirable characteristic in cookies—whereas cookies prepared with MAGs and RBW oleogels displayed hardness similar to those made with shortening. These findings suggest that direct‐method oleogels are better suited as shortening replacements in cookie preparation. However, the hardness of oleogels did not directly correlate with the hardness of cookies. This discrepancy may be attributed to the brittleness of indirect‐method oleogels, which stems from their rigid and discontinuous network structures. This brittleness correlated with poorer sensory acceptability and the higher hardness observed in cookies made with these oleogels. This behavior could be attributed to the different textural properties obtained by the different methodologies of preparation. Structured oils formed with low molecular weight gelators, typically associated with direct methods, rely on particle crystallization, where crystalline particles aggregate to form a network that immobilizes liquid oil. In contrast, indirect‐method oleogels form a three‐dimensional polymer network, that can result in greater brittleness.

In the domain of plastic fats, which are characterized by a semisolid state and spreadability at room temperature, several oil‐structuring approaches have been employed. For example, Sobolev et al. (2023) demonstrated that incorporating BW combined with HC in a 9:1 ratio resulted in a margarine formulation that was highly preferred by consumers when it contained 30% oleogel. Beyond that substitution level, the obtained products showed diminished sensory properties, mainly taste, odor, and consistency. A study by Cui et al. (2022) found that enzymatic interesterification of beef tallow and coconut oil produced margarine with a more compact texture and improved taste, smell, and overall acceptability when compared to traditional beef tallow‐based options.

In bakery applications, shortenings are essential for achieving desired textures in products such as pastries and cakes. Research shows that despite the differences between the fat alternative and the fat being replaced (e.g., palm oil, margarine, shortening), these differences do not always bring variation in the end product. The hot direct method where HPMC was used at a concentration of 1.5% in high‐oleic sunflower oil yielded croissants with lower saturated fat content and a texture profile similar to those made with commercial shortening, with no negative descriptors noted (Espert et al., 2023). Furthermore, Bascuas, Morell, et al. (2021) showed that combining HPMC with XG resulted in buns where no significant differences in texture were observed between those made with margarine and those made with oleogels; however, the buns exhibited a less porous structure. Other studies indicated that sponge cakes incorporating up to 50% peanut DAG oil–EC/MAG oleogel substitution maintained satisfactory texture profiles comparable to those made with commercial margarine. Due to the reduced firmness and chewiness of the cakes with 50% DAG‐based oleogels, higher mouthfeel, surface appearance, and elasticity acceptance scores were recorded during sensory assessment (Chen et al., 2023). Microcrystalline wax has also been recognized as a promising replacement for palm fat in baked goods like shortbread biscuits based on sensory evaluations. However, the samples with microcrystalline wax had a weaker aftertaste, raised fattiness, and a slightly firmer consistency (Schmid et al., 2020).

In the context of fats of animal origin, EC alongside sorbitan monostearate introduced via hot direct oleogelation showed minimal negative effects on sensory parameters when combined with various substrate oils like olive and linseed oil (Gómez‐Estaca et al., 2019). Similarly, Wang et al. (2024) found that the use of HPMC oleogels in cheese applications had minimal negative effects on sensory evaluations compared to control samples while improving spreadability and overall assessment. Moreover, Airoldi et al. (2022) demonstrated that ice cream made with a 50% fat replacement by CBW oleogel maintained similar acceptance levels as control samples. However, a complete replacement negatively affected the flavor by introducing floral and herbal descriptors. The use of HPMC oleogels also showed promise in replacing beef tallow while achieving lower cooking loss and softer textures, with the highest overall acceptability obtained at 50% replacement levels (Oh et al., 2019).

Cocoa butter plays a crucial role in providing the expected mouthfeel and glossy surface, alongside the texture and stability (resistance to fat‐bloom), to the chocolate products. Thus, cocoa butter substitutes are evaluated in such terms as the glossiness, melting sensation, and absence of the “waxy” mouthfeel, a typical effect of oil structuring. Sun et al. (2021) reported that oleogels created from β‐sitosterol and oryzanol exhibited enhanced glossiness and improved mouthfeel but reduced taste intensity compared to cocoa butter‐based chocolates. Additionally, Yılmaz and Öz (2024) found that combining SFW with polyglycerol stearate produced chocolates that had lower scores for shape and surface gloss but higher acceptability for flavor. Indirect oleogelation methods have shown promise as well. For example, Bascuas, Espert, et al. (2021) demonstrated that chocolate spreads incorporating 50% sunflower oleogel maintained attributes similar to control spreads regarding creaminess and cocoa flavor.

The mentioned studies further proved that oil structuring, through various innovative methods, not only allows for the development of healthier fat alternatives, maintaining the functional properties of fat ingredients, but also preserves the sensory qualities critical for consumer satisfaction across different food categories.

## 5 CHALLENGES OF OIL‐STRUCTURING METHODS

**FIGURE 6:**
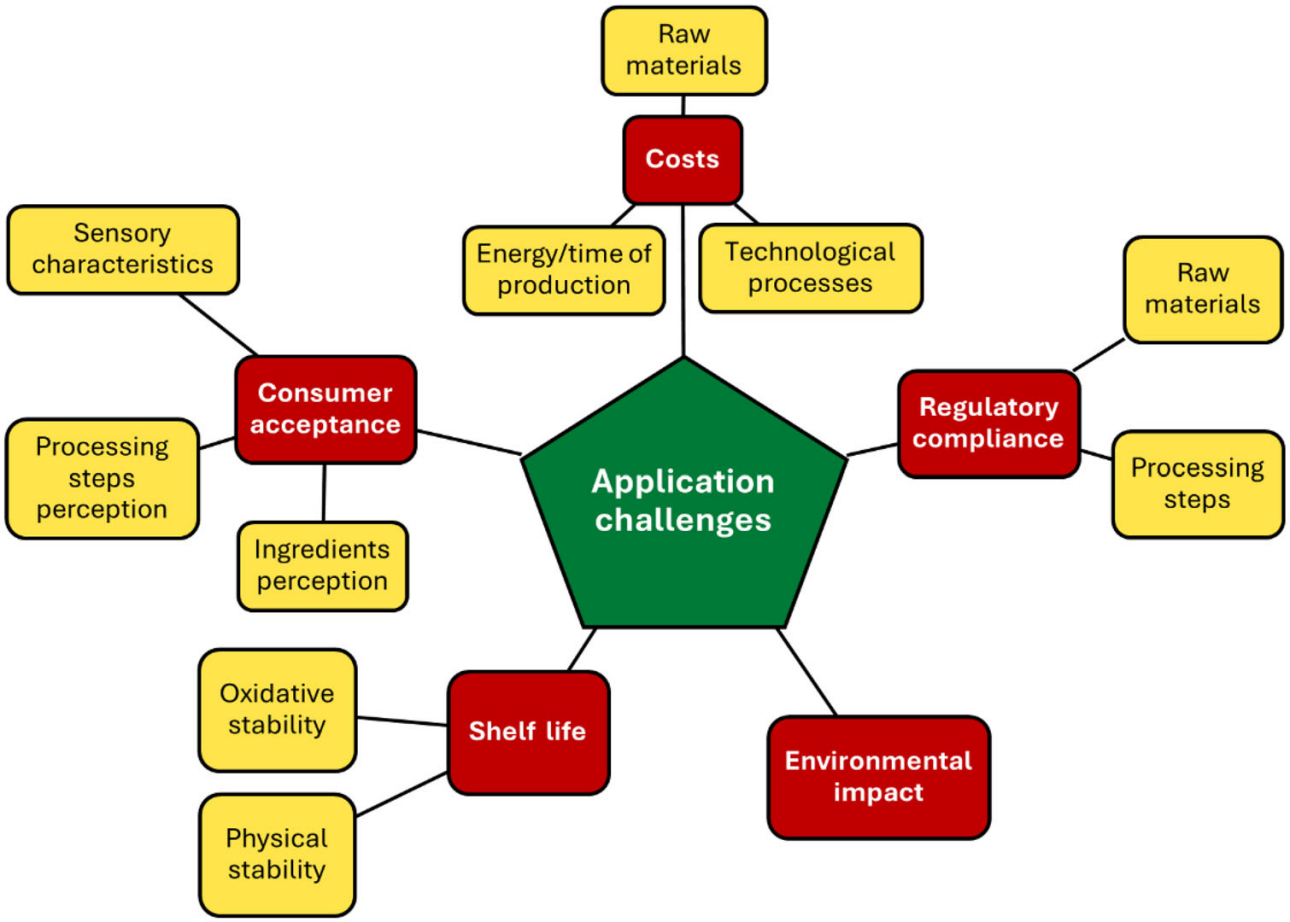
Challenges encountered in the industrial application of oils structured via various approaches.

Despite the vast differences between oil‐structuring methods, their application on the industrial scale faces a range of common challenges that must be considered as illustrated in Figure 6. These challenges encompass various aspects, including economic factors, regulatory compliance, environmental considerations, product stability, and consumer acceptance.

Technologies that are straightforward or closely related to conventional production methods may be adopted and used more quickly from a process standpoint. Interesterification‐based methods present challenges associated with the enzymes or chemicals used during the process. In the food industry, the chemical interesterification of fat blends is typically conducted at higher temperatures (100–110°C) for a relatively short time (typically a few minutes for randomization, with the complete reaction taking place in approximately 30 min) using metal alkylates or alkali metals as catalysts (Dijkstra & van Duijn, 2016). This process, distinguished by the random exchange of FA molecules, generates significant amounts of undesired byproducts (primarily soaps). Due to the high‐heat treatment, chemically interesterified oils (especially monounsaturated fatty acid [MUFA] and [PUFA]) are prone to oxidation, later forming volatile compounds that negatively affect the sensory properties. As a result, posttreatment steps like washing, bleaching, and deodorization are necessary. Moreover, chemical interesterification does not allow for partial reactions due to its rapid nature (Sivakanthan & Madhujith, 2020).

In enzymatic interesterification, depending on the enzyme used, specific positions can be targeted, offering higher selectivity and generating less undesirable byproducts compared to chemical interesterification. Enzymatic interesterification operates under mild conditions, resulting in minimal degradation of PUFAs. Additionally, biocatalysts are less toxic than chemical catalysts. Lipases provide catalytic versatility, being able to catalyze both hydrolysis and interesterification depending on the water content. They are highly stable in organic solvents and, at the same time, can be used in solvent‐free systems, which are often favorable for food applications. For these reasons, enzymatic interesterification boasts advantages such as easy product recovery, fewer processing steps, no *trans*‐FA production, high yield of natural fat, versatility in end‐product variety, and preservation of antioxidants like tocopherols (Sivakanthan & Madhujith, 2020). At the same time, a deodorization step is still required to remove unpleasant odors after the formation of peroxides, aldehydes, and ketones. In addition, a tedious control of the reaction conditions is required, such as temperature, pH, presence of heavy metals, and area of contact between the enzyme and the substrate oil. These are all factors affecting the enzyme efficiency. It should be also considered that the use of enzymes increases the price of the process, which varies from 3% to 10% (w/v) for industrial applications (Basso & Serban, 2019). However, the use of immobilized enzymes can decrease the costs. It has been shown that enzymes can be reused for up to three cycles without a loss of enzymatic activity and for up to eight cycles maintaining around 37% of their initial activity (Manzoor et al., 2024). In addition, when referring to the partial acylglycerols, which are produced in high amounts during glycerolysis, the ratios of resulting partial acylglycerides with different stereospecific positioning of the FA group within the molecule (1/3‐MAG, 2‐MAG, 1,2‐DAG, or 1,3‐DAG) can change over time due to the sensitivity to hydrolysis, the reactions of rearrangement, and inter‐ and intramolecular migration of acyclic groups (Younes et al., 2017). These changes can pose a stability issue when a certain species of partial acylglycerols is desired in the mixture.

From the regulatory compliance point of view, enzymatic treatment of lipids faces a bottleneck in the range of enzymes that can be used based on the EU Regulation on Food Enzymes (EC) No. 1332/2008 (European Union, 2008). In addition, enzymatic treatment must be mentioned on the label (Regulation [EU] No. 1169/2011 [European Union, 2011]). At the same time, several measures are taken to ease the application of enzymatic treatment for food manufacturers. Among them is the creation of a Union list of food enzymes to harmonize regulations across EU member states. Although this list has faced delays, its establishment must simplify the approval process and provide clarity on which enzymes are authorized for use. The procedure includes the safety evaluation by EFSA with subsequent approval from the European Commission and the inclusion to the list. While the Union list is not yet available, the Register of Food Enzymes to be considered for inclusion in the Union list can be accessed. Recent amendments to the regulation allow for grouping similar food enzymes under one application. This change can significantly reduce the administrative burden on companies seeking approval for the use of multiple enzymes (Regulation [EU] No. 1332/2008). In addition, the European Commission services have developed guidance documents to help food business operators determine whether a substance is qualified as a food enzyme or processing aid.

Direct oleogelation methods with their mild preparation conditions seem more promising as oleogels can be produced without altering the existing production lines. However, several challenges are also associated with the implementation of these methods. They include the storage instability of the produced oleogels, the low tolerance to shear force, the oxidation of essential FAs during production, and the high digestibility. For example, many oleogels made with low‐molecular‐weight gelators are shear‐thinning, losing viscosity when exposed to high‐shear conditions during the post‐addition process. The obtained product may then result in uneven oil distribution or oil leakage (Li et al., 2023). On the other hand, they are less prone to oxidation due to their lower heating temperatures with the exception of EC‐based oleogels, which definitely fall into this bottleneck.

From a sensory perspective, despite attempts to compensate for the absence of common fats by adjusting concentrations of gelators, the use of a single gelator may present issues, as an optimal network formation could be hard to achieve. It is worth underlining that the acceptance of wax in food products ranges within 3%–5% concentrations due to consumer preferences and food regulations. For example, CBW (E 903) has an acceptable daily intake (ADI) of 7 mg/kg bw/day according to the Expert Committee on Food Additives (JECFA) (Aguilar et al., 2012). To solve this issue, a patent (Marangoni et al., 2020) introduced a fat substitute utilizing wax blends or synergistic effects of multiple gelators, enhancing the network's physical properties while complying with food laws. However, concerns about the short‐term gel characteristics and long‐term stability persist. Conty et al. (2021) highlighted the diminishing hardness of oleogels over storage time, raising apprehensions regarding their prolonged shelf life and stability.

Finally, the applicability of direct methods is limited to molecules that are dispersible in oil and possess self‐assembly properties, which further restricts the range of oleogels that can be effectively produced. As large‐scale production will start, it has been anticipated that the prices of these gelators will decline. However, the approval and admission process for this novel food remains a task.

Indirect oleogelation methods show some unparalleled attractiveness thanks to the biomacromolecules’ unique properties. Although the production of such gels can theoretically be realized at an industrial scale using high‐energy emulsification and drying steps (Scharfe & Flöter et al., 2020), indirect preparation methods are still facing various challenges that limit their commercial uses. Specifically, when considering the oleogelation methods that require a drying step involving higher temperatures, particular attention should be given to the lipid oxidation issue. Furthermore, the precise control of process parameters is of utmost importance under real industrial conditions. Improper applications of shear forces, for example, can result in the disruption of droplet interfaces in emulsions, leading to significant issues (Feichtinger & Scholten, 2020).

One of the main challenges is found in the preparation steps, which are known to be more complex compared to the direct oleogelation alternatives. Sample preparation requires a broad set of specific instruments to obtain the desired final product. Published studies highlighted the need for different types of hand mixers, homogenizers, and even ultrasonic wave‐emitting devices for the foam formation step in the foam‐templated approach. Furthermore, other instruments are required for the following processing steps, such as a drying device to remove the water phase. A list of needed technologies has been provided by recent research, such as freeze‐drying, liquid nitrogen, or oven drying (Liu et al., 2017; Palla & Valoppi, 2024; Valoppi et al., 2024).

The preparation of structured oils, particularly through indirect oleogelation methods, requires not only a wide range of specialized instruments but also significant resources, including skilled personnel, time, and substantial initial investment.

From the regulatory compliance standpoint, oils structured via direct and indirect approaches fall under the Novel Food Regulation (Regulation [EU] No. 2015/2283 [European Union, 2015]). In case of uncommon food ingredients, such as waxes, producers must submit a dossier showing the product's safety to the EFSA. Similarly to the Union list of food enzymes, a consultation process on the novel food status is ongoing, which is designated to solve the uncertainties that food business operators might have regarding the applicability of the novel food. Starting from March 26, 2021, an E‐Submission Food Chain Platform has been introduced to facilitate the application process.

Considering these aspects, it is easy to understand why these oleogelation methods are not the most widespread compared to direct oleogelation. This is the reason why most of the reported studies have only been reproduced on a laboratory scale without providing input on how to efficiently adapt the process to an industrial scale. Thus, the application of these systems poses a challenge for manufacturing companies that are not willing to afford an investment without the certainty of obtaining a final product with the desired properties.

Challenges of indirect oleogelation methods are also linked to the final desired mechanical properties of the oleogels, which are highly dependent on the structuring agent (Valoppi et al., 2024). Thus, for a successful preparation of an oleogel, the ingredient selection is fundamental. For instance, in the emulsion‐based approach, different types of protein sources have been reported as efficient emulsifying agents to improve the emulsion's structure (plant‐based or non‐plant‐based). The different concentrations of these ingredients significantly affect the final mechanical properties (Espert et al., 2020). Additionally, other studies have reported the inclusion of sole or combined alternative ingredients, such as saponins (Chen & Yang, 2019; Li et al., 2023); vanillin and chitosan (Brito et al., 2022); flavanols (quercetin) and sodium caseinate (Santos et al., 2024); and tea polyphenol palmitate and citrus pectin (Luo et al., 2019), to improve the emulsification properties. Solvents (acetic acid or ethanol) can also improve the emulsion formation, although their addition may compromise the green label positioning of the food product or generate a negative response from the consumers’ acceptance.

Sustainability, scalability, and overall costs are critical considerations in oleogelation. Challenges such as the complexity of scaling up drying methods, lengthy preparation processes, and the sustainability limitations of certain systems further hinder industrial implementation. Consequently, manufacturing companies are hesitant to invest heavily without assurance of achieving a final product with the desired properties.

## 6 CONCLUSIONS AND PROSPECTS

In conclusion, even though structured oils have shown promising results as fat replacers in several food products, the fat‐to‐structured oil transition is still far. One of the main reasons is the gap of information regarding structured oils’ behavior under different storage conditions, their instability during processing, their comparison with alternative commercial shortenings, and their effects on actual application in different food products.

The production and use of structured oils have both potential benefits and challenges for long‐term environmental sustainability. On the positive side, they can utilize byproduct resources, replace less sustainable fats, and reduce food loss through improved stability. However, challenges include the environmental costs of raw material sourcing (e.g., deforestation for oil crops), energy‐intensive production processes, and waste management issues. To mitigate these impacts, adopting sustainable sourcing (e.g., algae or waste‐derived lipids), energy‐efficient production methods, and circular economy practices is essential. Advances in biotechnology offer promising pathways to reduce resource use and environmental footprints, positioning SLs as a sustainable option if managed responsibly.

The variability of structured oil properties, such as texture, mouthfeel, stability, and resistance to shear or heating, serves as an aide‐memoire indicating that considering only the physical properties of the “to‐be‐replaced fat” may not be the most effective strategy. There is no solution that “fits it all,” and thus, a prioritization must be followed. For example, in spreadable fats, such as margarine or butter alternatives, the formulation must ensure that the fat substitute can achieve the desired spreadability and stability at lower temperatures together with a melting sensation in the mouth. On the other hand, the physical properties of the fat, including resistance to intense shear and recrystallization after heat treatment, are desirable but not crucial factors to consider.

Similarly, in dairy products, fat substitute formulation must consider the unique emulsifying properties of dairy fats. For these products, instead, it is essential to consider the physical properties of the structured oils and their behavior during the different processing steps, such as pasteurization, homogenization, and fermentation.

Future studies should focus on the nutritional aspects related to the use of structured oil in food products. It is known that certain structured oils can contain dense lipids that are generally fully digestible, contributing to the risk of obesity and related diseases through potential overconsumption. A recent study has demonstrated that dietary oleogels can decrease body weight gain in rat models, indicating a potential benefit for weight management (Issara et al., 2020). Specifically, oleogel treatment has been shown to reduce high fat‐induced increases in lipid droplet size and hepatic steatosis area, suggesting a protective effect against fat accumulation in the liver. Furthermore, oleogel supplementation has been beneficial in suppressing adipogenesis and improving angiogenesis, which are crucial factors in metabolic health.

The primary health benefits of oleogels stem from their ability to replace *trans* and saturated fats with healthier unsaturated fats (Hwang, 2020). For instance, a study by Limpimwong et al. (2017) reported significant decreases in adipose tissue accumulation, total cholesterol in the liver, and TAG levels in the serum and liver among rats fed oleogel made with RBW compared to those fed beef tallow or commercial margarine. Additionally, oleogels have been shown to enhance the excretion of TAGs, total cholesterol, and bile acids in feces, indicating improved lipid metabolism.

Moreover, the oleogelators themselves may confer additional health benefits. For example, BW and CBW significantly decreased the lipogenesis pathway showing a pro‐obesogenic effect (Issara et al., 2019). In a study by Calligaris et al. (2020), it was found that the reduction of lipolysis in oleogels can be influenced by the type and amount of gelators used. The researchers showed that as the strength of the oleogel increased, the amount of lipolysis decreased (with plant sterols being the most effective, followed by RBW and MAGs). The reason for this difference was likely due to the structure of the gel network created by the gelators. Overall, these findings point out the potential of oleogels not only as healthier fat alternatives but also as functional ingredients that may positively influence lipid profiles and overall metabolic health.

The successful adoption of oleogelation technologies in food formulations is highly dependent on consumer perception, which is influenced by ingredient transparency, sensory attributes, and health benefits. While oleogels offer a promising alternative to traditional solid fats by reducing saturated fat content and eliminating *trans* fats, concerns regarding the use of structuring agents such as waxes and surfactants may limit acceptance. At the same time, market trends may reshape the consumer perception. Clean‐label trends emphasize the need for naturally derived oleogelators, such as plant‐based waxes, proteins, and dietary fibers, which are perceived as healthier and more sustainable. For example, according to 47% of German customers, if a peculiar ingredient is sustainable, they would be open to trying food or drink products that contain it (Schofield, 2024). Another study has shown that consumers tend to increase the purchase intention of oleogel‐containing products when aware of the added protein/fiber components in fat‐based products (Wang et al., 2024). Additionally, sensory characteristics, particularly texture and mouthfeel, play a crucial role in consumer preference, as some oleogels may impart waxy or greasy sensations that deviate from conventional fat‐based formulations. Optimizing the compositions of structured oils and blending them with conventional fats can improve sensory attributes and mimic the desirable properties of traditional lipids. Furthermore, positioning oleogels as functional ingredients with potential cardiovascular benefits, such as lowering saturated fat intake and improving lipid metabolism, may enhance consumer acceptance, following the recent consumer trends on healthier nutrition profiles and “prescription food” (Schofield, 2024). Effective communication strategies, including clear labeling and consumer education, will be essential in promoting the benefits of oleogels in reformulated food products. Future research should focus on consumer behavior studies and sensory optimization to facilitate the integration of oleogelation in commercially viable food applications.

Recent advancements in artificial intelligence (AI) suggest that integrating this technology into the research and production of structured oils could not only facilitate processes but also catalyze innovation in creating healthier food options. One example of how AI can enhance various aspects of food ingredient innovation is the discovery of new ingredients and optimizing product development processes. For instance, Shiru's OleoPro, a fat replacer developed using the AI platform Flourish, significantly reduces SFA content in alternative protein foods by up to 90% (Schofield, 2024). Additionally, in the context of structured oil analysis, Palla and Valoppi (2024) highlight the potential of machine learning to enhance image analysis, enabling researchers to investigate the nano, micro, and macro characteristics of oleogels more effectively. Advanced microscopy techniques combined with machine learning could provide detailed quantitative data on properties like crystal size, porosity, and crystallization kinetics (Palla & Valoppi, 2024). Furthermore, real‐time monitoring techniques utilizing supervised machine learning have been developed to enhance lipid crystallization processes, providing valuable insights that can drive efficiency in food manufacturing (Metilli et al., 2022).

Overall, the findings published so far show that oil structuring can be considered a valuable approach for new product development. By applying a comprehensive approach, which would consider all the abovementioned challenges and prioritize the crucial aspects of product development, the potential of oil structuring will be fully exploited. Ongoing research and development are needed to address these challenges and unlock the full potential of oil structuring in the food industry.

## AUTHOR CONTRIBUTIONS

**Ecaterina Savchina**: Conceptualization; investigation; writing—original draft; visualization; writing—review and editing. **Antonella L. Grosso**: Conceptualization; investigation; writing—original draft. **Petra Massoner**: Writing—review and editing; funding acquisition. **Ksenia Morozova**: Writing—review and editing; visualization. **Matteo M. Scampicchio**: Writing—review and editing; supervision; resources; visualization. **Giovanna Ferrentino**: Conceptualization; visualization; writing—review and editing; project administration; supervision.

## CONFLICT OF INTEREST STATEMENT

The authors declare no conflicts of interest.
